# Comparative efficacy and safety of different SGLT2 inhibitor-based combination strategies in HFrEF: a systematic review and network meta-analysis

**DOI:** 10.3389/fendo.2025.1742128

**Published:** 2026-01-02

**Authors:** Neng Jiang, Yuling Zhang, Yue Tang, Hongmei Tan

**Affiliations:** 1 School of Medicine, Sun Yat-Sen University, Shenzhen, Guangdong, China; 2 Department of Cardiovascular Medicine, Sun Yat-sen Memorial Hospital, Sun Yat-sen University, Guangzhou, Guangdong, China; 3 Department of Cardiovascular Medicine, The Seventh Affiliated Hospital, Sun Yat-sen University, Shenzhen, Guangdong, China

**Keywords:** cardiovascular death, drug combinations, heart failure with reduced ejection fraction, network meta-analysis, sodium-glucose transporter 2 inhibitors

## Abstract

**Background:**

The optimal combination therapy for heart failure with reduced ejection fraction (HFrEF) involving sodium-glucose transporter 2 inhibitors (SGLT2i), angiotensin receptor–neprilysin inhibitors (ARNI), and conventional triple therapy remains uncertain due to the lack of direct comparative evidence.

**Methods:**

We conducted a Bayesian network meta-analysis of randomized controlled trials (RCTs) comparing six treatment regimens. Primary outcome was the composite of cardiovascular death and hospitalization. Secondary outcomes included all-cause mortality, Kansas City Cardiomyopathy Questionnaire (KCCQ) scores, 6-minute walk distance (6MWD), N-terminal pro-B-type natriuretic peptide (NT-proBNP) levels, and adverse events (hypotension, hyperkalemia, renal events). All analyses were performed under a Bayesian statistical framework, and the relative efficacy and safety of the six regimens were ranked using surface under the cumulative ranking curve (SUCRA) probabilities.

**Results:**

A total of 22 studies (21 RCTs) involving 24,499 patients were included. For the primary composite outcome, Sotagliflozin-based quadruple therapy ranked first (SUCRA: 90.8%; OR: 0.49, 95%CI: 0.16 to 1.47). Dapagliflozin+triple therapy (SUCRA: 76.8%; OR: 0.83, 95%CI: 0.69 to 0.98) and ARNI+BB+MRA (SUCRA: 76.6%; OR: 0.83, 95%CI: 0.75 to 0.92) demonstrated significant reductions in all-cause mortality. ARNI-based triple therapy was associated with a significantly increased risk of hypotension (OR: 1.67, 95%CI: 1.48 to 1.90), whereas Dapagliflozin and Empagliflozin showed protective effects against renal adverse events. No regimen significantly increased hyperkalemia risk. Additionally, a statistically significant interaction was observed between treatment effects and baseline diabetes burden for the primary outcome (*p* = 0.003).

**Conclusions:**

This network meta-analysis demonstrates that while all quadruple therapy regimens improve outcomes in HFrEF, important distinctions exist. Sotagliflozin-based therapy may offer advantages in preventing hospitalizations, whereas Dapagliflozin and ARNI are comparable for mortality reduction. The safety profiles differ significantly, particularly regarding hypotension risk with ARNI and renal protection with SGLT2i. The choice of regimen should be individualized based on patient priorities, comorbidities, and safety tolerability profiles.

**Systematic Review Registration:**

https://www.crd.york.ac.uk/PROSPERO/view/CRD420251112344

, identifier CRD420251112344.

## Graphical abstract

**Figure:**
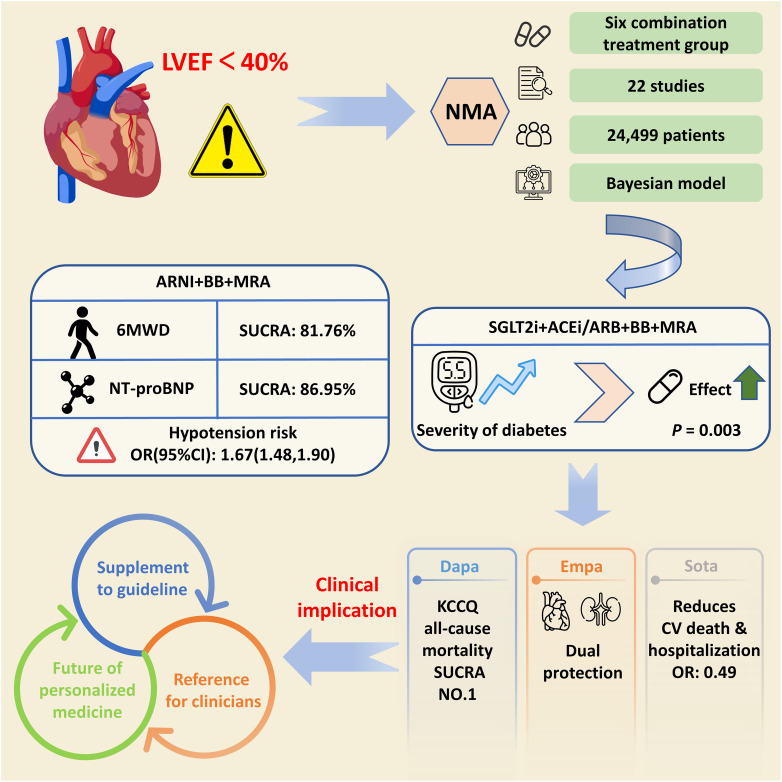
Network meta-analysis of six therapeutic regimens in HFrEF: main results and clinical implications. Graphic summary of NMA on pharmacotherapies for HFrEF, highlighting key findings and clinical implications. ACEi, angiotensin-converting enzyme inhibitor; ARB, angiotensin receptor blocker; ARNI, angiotensin receptor neprilysin inhibitor; BB, beta-blocker; CV, cardiovascular; Dapa, Dapagliflozin; Empa, Empagliflozin; LVEF, left ventricular ejection fraction; MRA, mineral receptor antagonist; NMA, network meta-analysis; Sota, Sotagliflozin; SUCRA, surface under the cumulative ranking curve.

## Highlights

**What is currently known about this topic?**

- HFrEF has high morbidity and mortality worldwide.
- Guidelines recommend quadruple therapy for HFrEF.
- Optimal combination regimens remain uncertain.

**What is the key research question?**

- How do SGLT2 inhibitor-based combination regimens compare in efficacy and safety for HFrEF?

**What is new?**

- Sotagliflozin-based therapy ranks best for CV death and hospitalization.
- Dapagliflozin and ARNI reduce all-cause mortality effectively.
- Safety profiles differ: ARNI increases hypotension; SGLT2i protect kidneys.

**How might this study influence clinical practice?**

- Enables personalized HFrEF therapy based on patient priorities and risks.

## Introduction

Heart failure with reduced ejection fraction (HFrEF), defined as symptomatic heart failure with a left ventricular ejection fraction (LVEF) ≤40% (1), continues to pose a formidable challenge to global public health systems due to its high rates of morbidity and mortality, as well as substantial healthcare expenditures. Epidemiological studies report an estimated worldwide prevalence approaching 26 million individuals, with 5-year mortality rates exceeding 50% even in the context of considerable therapeutic innovations (2). Clinically, the trajectory of HFrEF is marked by recurrent hospitalizations for acute decompensations and a progressive deterioration in functional status (3).

International clinical guidelines, most notably the 2022 updated joint recommendations from the American Heart Association, American College of Cardiology, and Heart Failure Society of America (AHA/ACC/HFSA) alongside the 2021 guidelines issued by the European Society of Cardiology (ESC), unanimously advocating for a foundational quadruple pharmacological regimen (1, 4). This regimen consists of four key drug classes: renin-angiotensin-aldosterone system inhibitors (RAASi, including angiotensin-converting enzyme inhibitors [ACEi], angiotensin receptor blockers [ARB], and angiotensin receptor-neprilysin inhibitors [ARNI]), beta-blockers (BB), mineralocorticoid receptor antagonists (MRA), and sodium-glucose cotransporter-2 inhibitors (SGLT2i). This consolidated strategy marks a significant evolution in standard practice, with trial data indicating a substantial 30–40% decline in cardiovascular mortality, supporting the premise that multimechanistic pharmacologic intervention can modify the clinical trajectory of HFrEF (5).

Despite these advances, numerous areas of clinical ambiguity persist, particularly regarding the optimal selection of combination regimens. First, there is a lack of direct comparisons to determine the optimal choice between adding SGLT2i or using ARNI on the basis of the standard triple therapy (ACEi/ARB+BB+MRA) for HFrEF (6). Second, clinical evidence is insufficient to distinguish the efficacy and safety distinctions within the SGLT2 inhibitor class, specifically among Dapagliflozin, Empagliflozin, and Sotagliflozin. Third, the tolerability of intensive multidrug regimens necessitates careful scrutiny, especially concerning the potential for renal impairment, hyperkalemia, and hypotensive episodes in vulnerable subgroups, such as patients with underlying chronic kidney disease or diabetes mellitus.

Traditional pairwise meta-analyses have validated the efficacy of individual drug classes in HFrEF but are limited in their ability to address comparative questions across multiple therapeutic regimens, as they cannot synthesize indirect evidence from overlapping trial networks (7). A recently published network meta-analysis (NMA) has underscored the therapeutic potential of drug combinations in HFrEF management (8). However, by focusing narrowly on clinical symptoms of cardiac reverse remodeling (CCR) endpoints and not making distinctions regarding the effects brought about by the different types of SGLTi, it fails to fully capture the multifaceted burden of HFrEF and the diverse therapeutic options available. Thus, to provide a more comprehensive understanding of the comparative efficacy and safety of different combination regimens, we conducted a network meta-analysis.

A key assumption underlying the validity of NMA is transitivity, which implies that studies included in the network can be compared indirectly because they are sufficiently similar in all effect modifiers besides the interventions being compared. To ensure the plausibility of this assumption, we pre-specified criteria for clinical and methodological similarity. Clinically, we limited our analysis to studies enrolling adult patients with HFrEF (LVEF ≤40%) and ensured that the distribution of potential effect modifiers was comparable across different treatment comparisons. Methodologically, we included only randomized controlled trials (RCTs) with a minimum follow-up of 8 weeks and conducted a rigorous risk of bias assessment. In this study, we integrated all available evidence on six key therapeutic regimens for HFrEF, evaluating nine critical endpoints, aims to guide clinical decisions from three aspects: therapeutic efficacy, safety risks, and biomarkers.

## Methods

### Protocol and registration

This network meta-analysis was prospectively registered in the International Prospective Register of Systematic Reviews (PROSPERO; registration ID: CRD420251112344) before initiating data synthesis and this study was conducted in full accordance with the pre-registered protocol. The manuscript provides detailed operational definitions and statistical specifications (use of Bayesian framework, Markov Chain Monte Carlo [MCMC] parameters) that implement the pre-specified analytical plan. The comprehensive protocol, meticulously crafted in adherence to the Preferred Reporting Items for Systematic Reviews and Meta-Analyses for Network Meta-Analyses (PRISMA-NMA) guidelines, delineates key components including systematic search strategies, eligibility criteria, and analytical approaches (9).

### Eligibility criteria

#### Inclusion criteria

Studies were selected following the PICOS framework to maintain methodological rigor. For the population, we included adult patients (≥18 years) with HFrEF, defined as symptomatic heart failure with left ventricular ejection fraction ≤40% regardless of etiology. Regarding interventions & comparators, six predefined regimens were chosen: (1) ACEi/ARB + BB + MRA; (2) Dapagliflozin + ACEi/ARB + BB + MRA; (3) ARNI + BB + MRA; (4) Empagliflozin + ACEi/ARB + BB + MRA; (5) ACEi/ARB + BB; (6)Sotagliflozin + ACEi/ARB + BB + MRA.

The grouping or separation of drug classes into distinct nodes follows a pre-specified evidence hierarchy integrating clinical guidelines, pharmacological mechanisms, and clinical trial data. ACEi and ARB are clustered into a single “ACEi/ARB” node. This grouping is justified by their interchangeability in clinical guidelines: for HFrEF management, guidelines from bodies like AHA/ACC/HFSA and ESC recommend ACEi or ARB as equivalent foundational components of RAAS inhibition when ARNI is not used (1, 4). They share a core therapeutic target of inhibiting the RAAS and produce comparable hemodynamic and clinical effects in HFrEF with no large-scale randomized trials demonstrating one class’s superiority over the other for HFrEF mortality or hospitalization outcomes. In contrast, the SGLT2 inhibitors Dapagliflozin, Empagliflozin, and Sotagliflozin are analyzed as separate nodes. This distinction arises from their distinct pharmacological profiles: Sotagliflozin is a dual SGLT1 and SGLT2 inhibitor, while Dapagliflozin and Empagliflozin are selective SGLT2 inhibitors. Additionally, they also hold distinct regulatory and clinical status: each gained approval via separate pivotal trials, and clinicians commonly select specific agents within the class in real-world practice. This node-definition approach balances clinical relevance and methodological rigor, aligning with real-world treatment decision-making and the current evidence base. We recognize that the included trials were not originally designed to compare these specific, multi-drug regimens. Our approach to node definition is a pragmatic and necessary simplification to enable quantitative synthesis using NMA. The primary goal is to model the incremental value of adding a specific agent (e.g., an SGLT2i or ARNI) to a foundational background therapy, which aligns with the clinical question of how to optimize GDMT in a stepwise manner.

For outcomes, the primary was the composite of cardiovascular (CV) death and hospitalization. Secondary outcomes included dichotomous (all-cause mortality; hypotension; hyperkalemia; renal adverse events) and continuous (KCCQ-TSS, KCCQ-CS mean change from baseline; 6MWD; NT-proBNP mean change from baseline) measures. Each outcome measure was defined with strict rigor. Detailed definitions are available in the study protocol registered on the PROSPERO platform. Study design encompassed randomized controlled trials with follow-up ≥8 weeks.

#### Exclusion criteria

Studies were excluded if they met any of the following: (1) Patients with HFrEF who are under 18 years old, or pregnant individuals; (2) non-RCT study designs; (3) Trials with incomplete outcome reporting or unextractable data.

### Assessment of transitivity and consistency

The validity of indirect comparisons in an NMA relies on the transitivity assumption. We assessed the plausibility of transitivity by examining the distribution of clinically important effect modifiers across the available treatment comparisons. These modifiers included mean age, mean LVEF, and proportion of male participants. The distributions of these variables across studies forming the network were compared visually and are summarized in a new supplementary table (**Supplementary Table S1**). No major imbalances were identified that would violate the transitivity assumption.

Furthermore, we evaluated the statistical inconsistency in the network whenever possible. Global inconsistency was assessed using the design-by-treatment interaction model. Local inconsistency for closed loops was evaluated using the node-splitting method, which examines disagreement between direct and indirect evidence for a specific treatment comparison. A p-value ≥ 0.05 in the inconsistency test was considered to indicate no significant inconsistency.

### Information sources and search strategy

Comprehensive searches were performed across multiple sources: electronic databases (PubMed, EMBASE, Cochrane library, Web of Science) from inception to July 10^th^ 2025. The searches for each database were peer-reviewed using the PRESS checklist. The complete, executable search strategies for all databases are provided in **Supplementary Methods: Search Strategy**. Additionally, we manually screened the reference lists of all included studies and relevant systematic reviews to identify any potentially missed publications.

### Study selection and data collection process

The study selection process was conducted by two independent investigators with expertise in cardiology systematic reviews. Using a predefined protocol, investigators initially screened titles and abstracts against eligibility criteria in the ZOTERO software. Potentially relevant records were retrieved for full-text assessment. Disagreements were resolved by discussion, or if necessary, consulting the third researcher. A standardized data extraction form was used to collect the following information from each included study: first author name, year of publication, total sample size, details of interventions and comparators, duration of follow-up, and baseline patient characteristics (including sex distribution, mean age, mean LVEF, the proportion of participants using ACEi/ARB/ARNi as combination drugs, the proportion of participants using BB as combination drugs, and the proportion of participants using MRA as combination drugs). Outcome data were extracted in their original format to preserve analytical integrity. For continuous variables, data were captured as means with standard deviations (SD) as reported. For dichotomous outcomes, both event counts and total sample sizes per group were recorded to enable calculation of effect sizes. When raw data were unavailable or ineligible formats, values were converted using validated conversion methods consistent with systematic review guidelines.

To ensure consistency in defining therapeutic regimens and accounting for background therapies, we adopted a systematic approach to classify concomitant medications, informed by prior methodological standards (10). Treatments were categorized by drug-group combinations based on the 50% patient threshold. In other words, if more than 50% of participants in an arm received specific concomitant drugs, the treatment was defined as a combination therapy (core study drug group + combination drug group). This approach accounts for both the primary interventions and background therapies that could influence treatment effects in HFrEF patients, overcoming the limitations of some previous analytical articles. In addition, diuretics were excluded from concomitant therapy classification if not indicated for MRA-related purposes (8).

### Risk of bias assessment

Risk of bias (RoB) assessment was performed with the Cochrane risk of bias tool, covering five key domains: (1)Bias from randomization (random sequence generation, allocation concealment); (2)Bias due to intervention deviations; (3)Bias from missing outcome data; (4)Bias in outcome measurement; (5)Bias from selective outcome reporting. Each domain was rated as “low risk of bias”, “unclear risk of bias”, or “high risk of bias” (11, 12). Two researchers independently conducted the assessment. Discrepancies were resolved via discussion or third-researcher adjudication. RoB results were visualized using RevMan 5.4(Cochrane Collaboration, London, UK).

### Data analysis

All statistical analyses were conducted using R Studio version 4.5.1 (R Core Team, Vienna, Austria) and Stata 18 (StataCorp). For this network meta-analysis, the gemtc package was employed to implement Bayesian models. For outcome measures, continuous variables were analyzed using the mean difference (MD) with corresponding 95% confidence intervals (CIs), whereas dichotomous variables were evaluated using the risk ratio (RR) or odds ratio (OR) (selected based on outcome nature) with 95% CIs—both metrics served as effect size indicators.

In the Bayesian NMA framework, the Markov chain Monte Carlo (MCMC) method was used to estimate posterior distributions, with a step size of 10 set for sampling efficiency. Specifically, a four-chain Gibbs sampling approach was adopted, consisting of 10,000 burn-in iterations followed by 200,000 sampling iterations for parameter estimation. To evaluate network consistency, node analysis models were applied to detect potential inconsistency. P-values were collected from the inconsistency tests: a P-value < 0.05 indicated statistically significant inconsistency, whereas a P-value ≥ 0.05 justified the use of the consistency model.

The surface under the cumulative ranking curve (SUCRA) was utilized to rank the relative efficacy of all treatments for each outcome; SUCRA values range from 0% to 100%, with higher values indicating a greater likelihood of being the most effective treatment.

### Model selection and data synthesis

The choice between fixed-effects and random-effects models was pre-specified based on a hierarchical framework that considered both clinical expectations and statistical findings.

#### Primary model

Given the anticipated clinical and methodological heterogeneity across trials (e.g., variations in follow-up duration, background guideline-directed medical therapy, and patient population severity), the random-effects model was designated as the primary analysis for all outcomes. This model accounts for the assumption that the true treatment effects may vary across studies and provides a more conservative estimate when heterogeneity is present.

#### Fixed-effects model application

A fixed-effects model was pre-specified to be used only under the following two conditions: a. If the number of studies in a treatment network was very small (e.g., fewer than 5 studies), making the estimation of the between-study variance (τ²) unreliable. b. If the statistical heterogeneity was found to be negligible (*I²* = 0%) and a compelling clinical rationale existed to support effect homogeneity across the included studies (e.g., all studies had nearly identical protocols and patient populations).

Heterogeneity was assessed using the *I²* statistic, where *I²* > 50% was considered to represent substantial heterogeneity. The between-study variance (τ²) was also estimated. For outcomes where both models were applicable, a sensitivity analysis was performed by comparing the results from both fixed-effects and random-effects models to assess the robustness of the conclusions.

## Result

**Figure 1:**
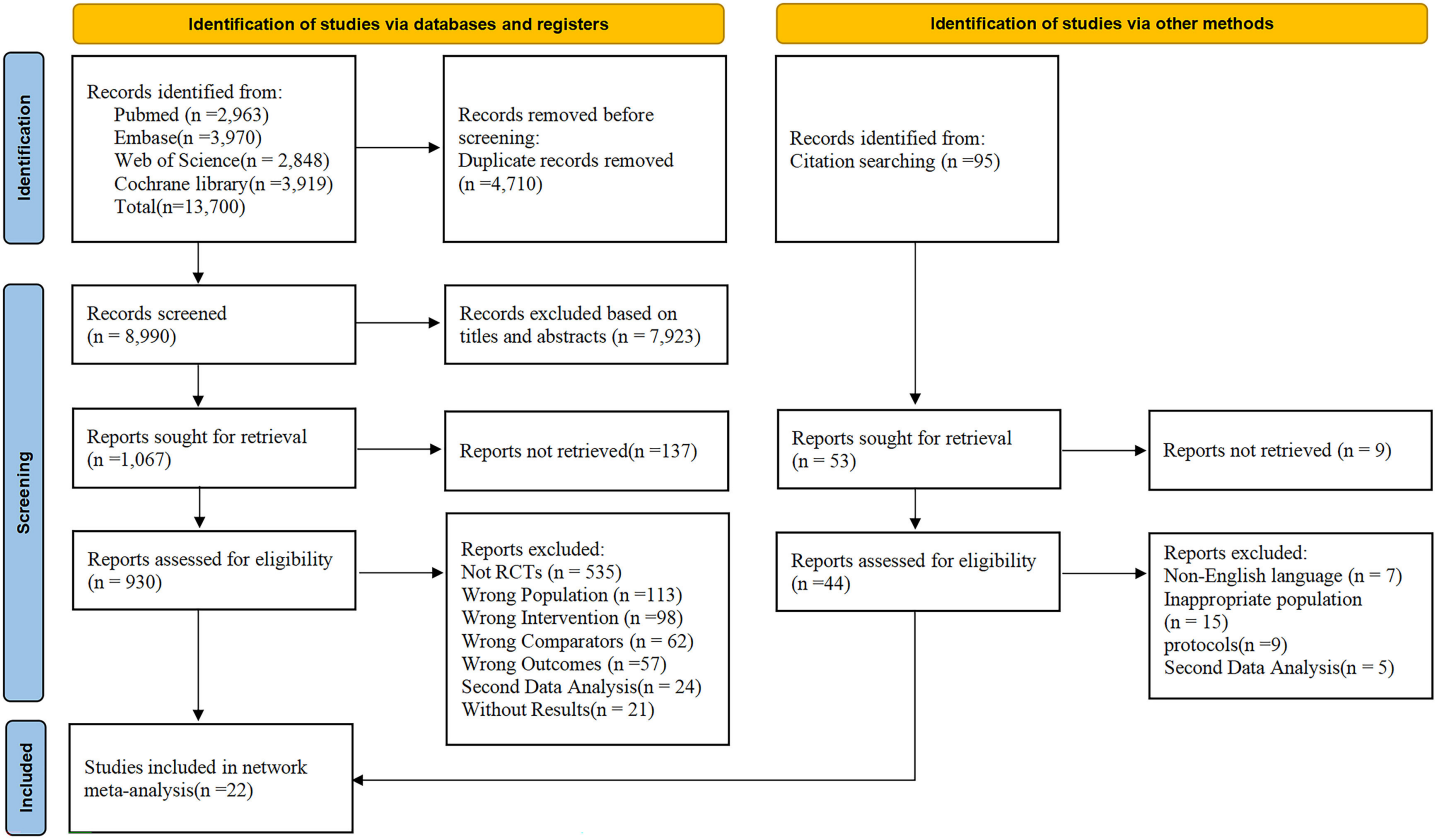
PRISMA flowchart. An overview of study selection process. RCT, randomized controlled trial.

An initial database search retrieved 13,700 studies from Pubmed, Embase, Web of Science and Cochrane library. After removing 4,710 duplicates, 7,923 studies were excluded via title - abstract screening, and another 1,047 were excluded following full-text review. Ultimately, 22 studies (21RCTs) (13–34) were eligible for analysis (**Figure 1**).

### Study characteristics and quality

22 studies cover 21 RCTs because two studies reported the same RCT, comprising 24,499 patients with HFrEF. The baseline characteristics of the included studies and patient populations are summarized in **Supplementary Table S1**. The mean age across studies ranged from 58 to 70.5 years, the mean LVEF ranged from 26% to 32%, and the proportion of patients with diabetes varied from 0% to 100%, with the majority of studies falling between 30% and 60%. This comparability in key patient characteristics supports the transitivity assumption for the network meta-analysis. **Supplementary Figure S1** showed the result of bias risk assessment.

### CV death and hospitalization

**Figure 2:**
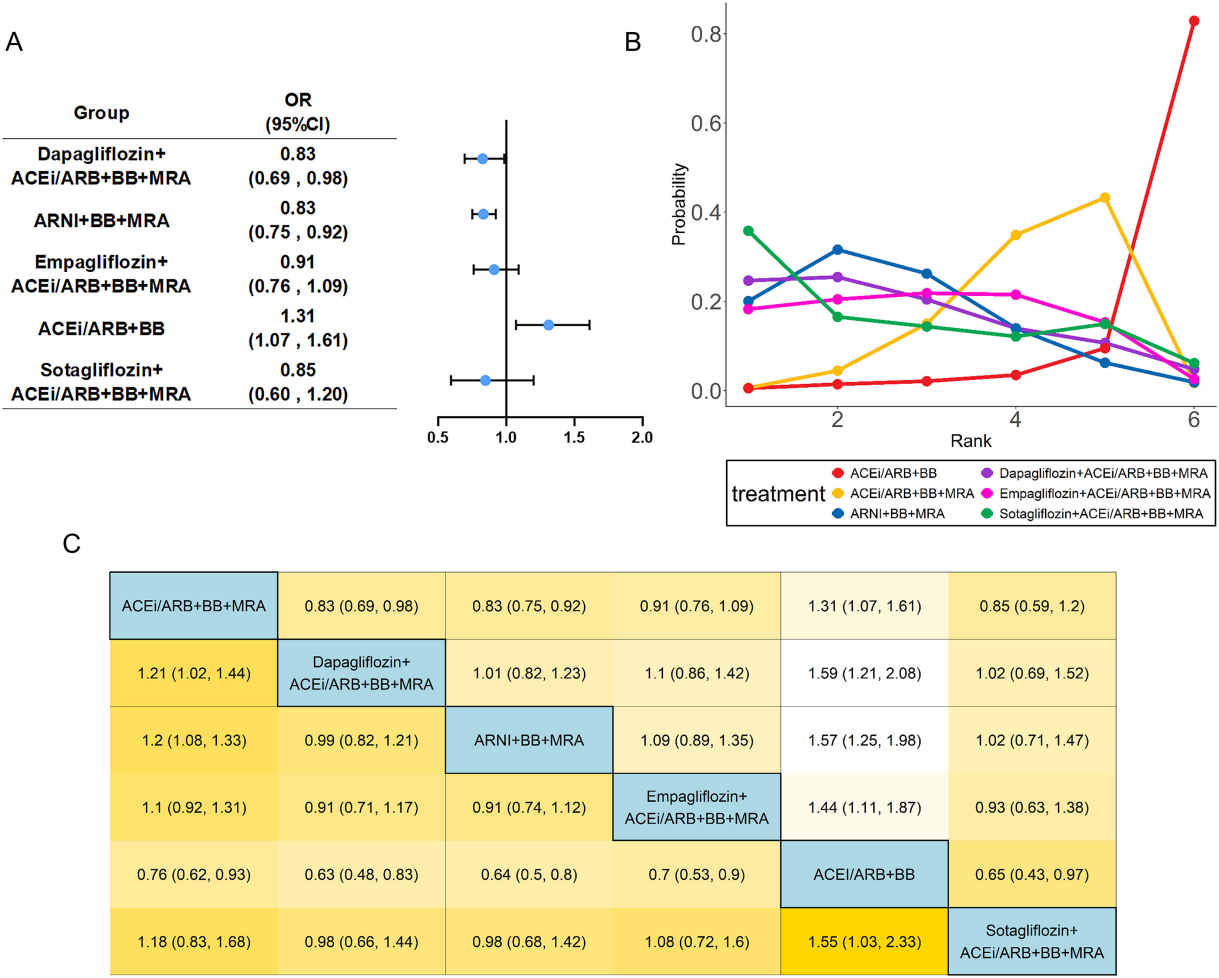
Network meta-analysis of CV death and hospitalization. The visualization of the results includes **(A)** forest plots, **(B)** rank probability plot, and **(C)** league table. ACEi, angiotensin-converting enzyme inhibitor; ARB, angiotensin receptor blocker; ARNI, angiotensin receptor neprilysin inhibitor; BB, beta-blocker; MRA, mineral receptor antagonist.

Fifteen studies (13–17, 21–24, 26, 27, 30, 31, 33, 34) evaluated the composite endpoint of CV death and hospitalization, including 23,203 patients with HFrEF and six treatments. Specific group information of combination medication was shown in a network map (**Supplementary Figure S2A**). The common control group was defined as the traditional triple drug combination (ACEi/ARB+BB+MRA). In the analysis of the composite outcome of CV death and hospitalization, Sotagliflozin-based quadruple therapy demonstrated the highest probability of being the best treatment (SUCRA: 90.8%) (**Table 1**). The point estimate suggests a substantial 51% reduction in the odds of the composite endpoint (OR: 0.49). However, the wide confidence interval (95% CI: 0.16 to 1.47) indicates uncertainty, as it includes the possibility of both a large benefit and no effect (**Figure 2**). Dapagliflozin-based quadruple therapy (SUCRA: 68.1%; OR: 0.77, 95% CI: 0.40 to 1.81) and ARNI-based triple therapy (SUCRA: 76.6%; OR: 0.93, 95% CI: 0.59 to 1.60) both demonstrated a trend toward reduction in outcomes. Although statistical significance was not achieved (as their 95% CIs crossed 1.0), the observed effect trends and their SUCRA rankings are considered clinically relevant in the context of heart failure trials.

**Table 1:** SUCRA comprehensive ranking of six therapeutic regimens.

Treatment	CV death and hospitalization	All cause mortality	6MWD	KCCQ-TSS	KCCQ-CS	NT-proBNP	Hypo- tension	Hyper- kalemia	Renal adverse events
ACEi/ARB+ BB+MRA	42.81%	26.92%	25.84%	27.92%	1.92%	21.17%	73.10%	35.99%	17.39%
Dapagliflozin+ ACEi/ARB+ BB+MRA	68.11%	76.82%	37.04%	91.28%	84.86%	51.53%	52.56%	70.98%	86.17%
ARNI+ BB+MRA	50.96%	76.34%	81.76%	45.06%	56.19%	86.95%	11.74%	24.23%	39.75%
Empagliflozin+ ACEi/ARB+ BB+MRA	43.79%	52.31%	55.36%	76.62%	57.02%	40.36%	36.58%	44.59%	86.94%
ACEi/ARB+ BB	3.52%	0.52%	/	9.12%	/	/	87.61%	79.79%	18.38%
Sotagliflozin+ ACEi/ARB+ BB+MRA	90.81%	67.10%	/	/	/	/	38.42%	44.41%	51.37%

Summary table of SUCRA comprehensive rankings for six therapeutic regimens across nine clinical outcomes, to facilitate comparison of their relative effectiveness. ACEi, angiotensin-converting enzyme inhibitor; ARB, angiotensin receptor blocker; ARNI, angiotensin receptor neprilysin inhibitor; BB, beta-blocker; CV, cardiovascular; KCCQ-CS, Kansas City Cardiomyopathy Questionnaire Clinical Summary Score; KCCQ-TSS, Kansas City Cardiomyopathy Questionnaire Total Symptom Score; MRA, mineral receptor antagonist; NT-proBNP, N-terminal pro-B-type natriuretic peptide; 6MWD, 6-minute walk distance.

**Figure 3:**
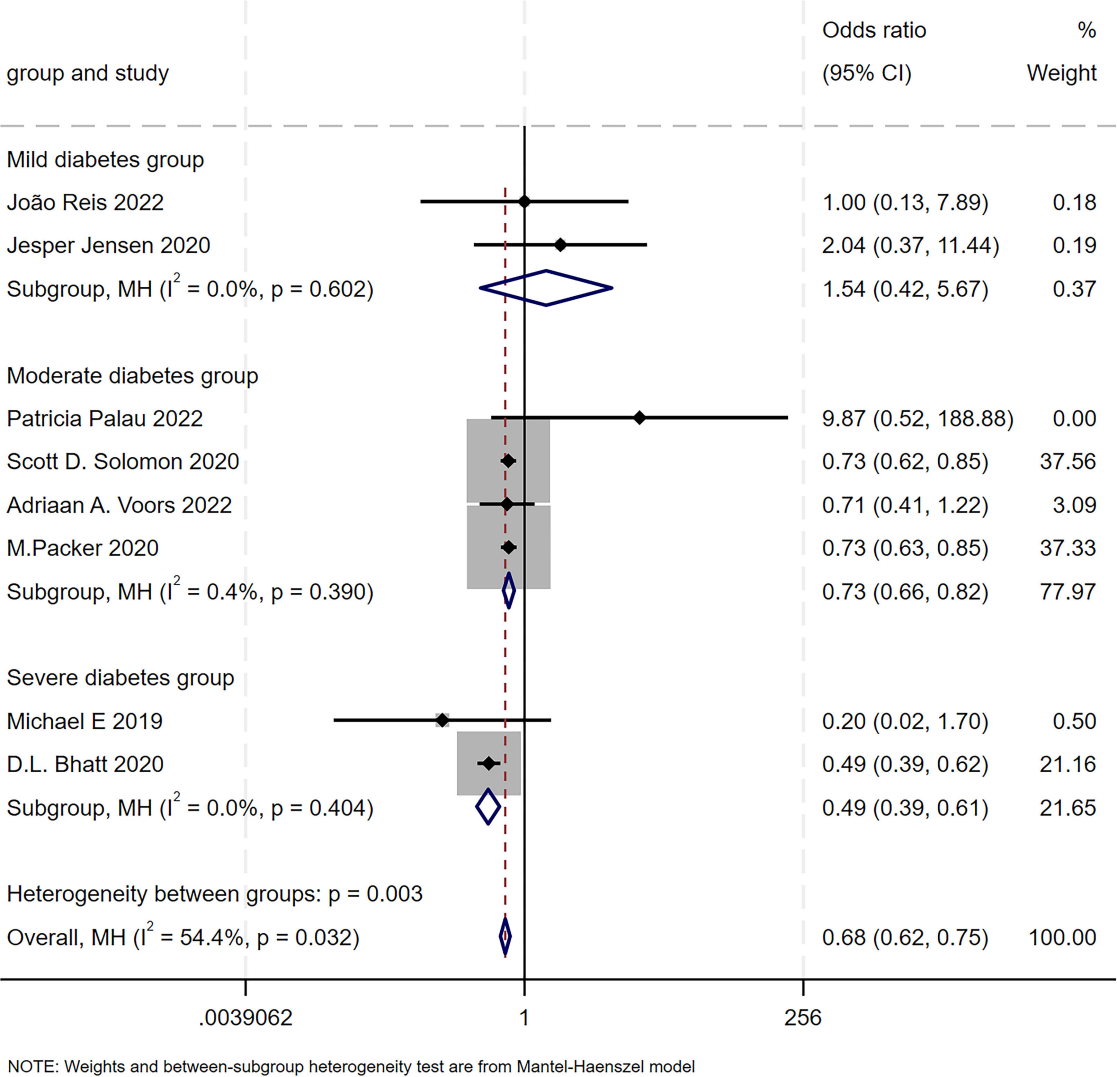
Subgroup analysis of SGLT2i-based quadruple therapy by diabetes severity groups. Forest plot for CV death and hospitalization across subgroups stratified by diabetes severity.

A subgroup analysis was performed to evaluate the treatment effect on the composite outcome of cardiovascular death and hospitalization based on the prevalence of diabetes within the study population (**Figure 3**). Studies were categorized into three subgroups: a mild diabetes burden subgroup (where <30% of the study participants had diabetes), a moderate diabetes burden subgroup (30% to 60% prevalence of diabetes), and a severe diabetes burden subgroup (>60% prevalence of diabetes). The analysis revealed a statistically significant interaction between the treatment effect and the baseline diabetes burden subgroup (test for subgroup differences: *p* = 0.003) and demonstrated negligible heterogeneity within subgroups.

### All-cause mortality

**Figure 4:**
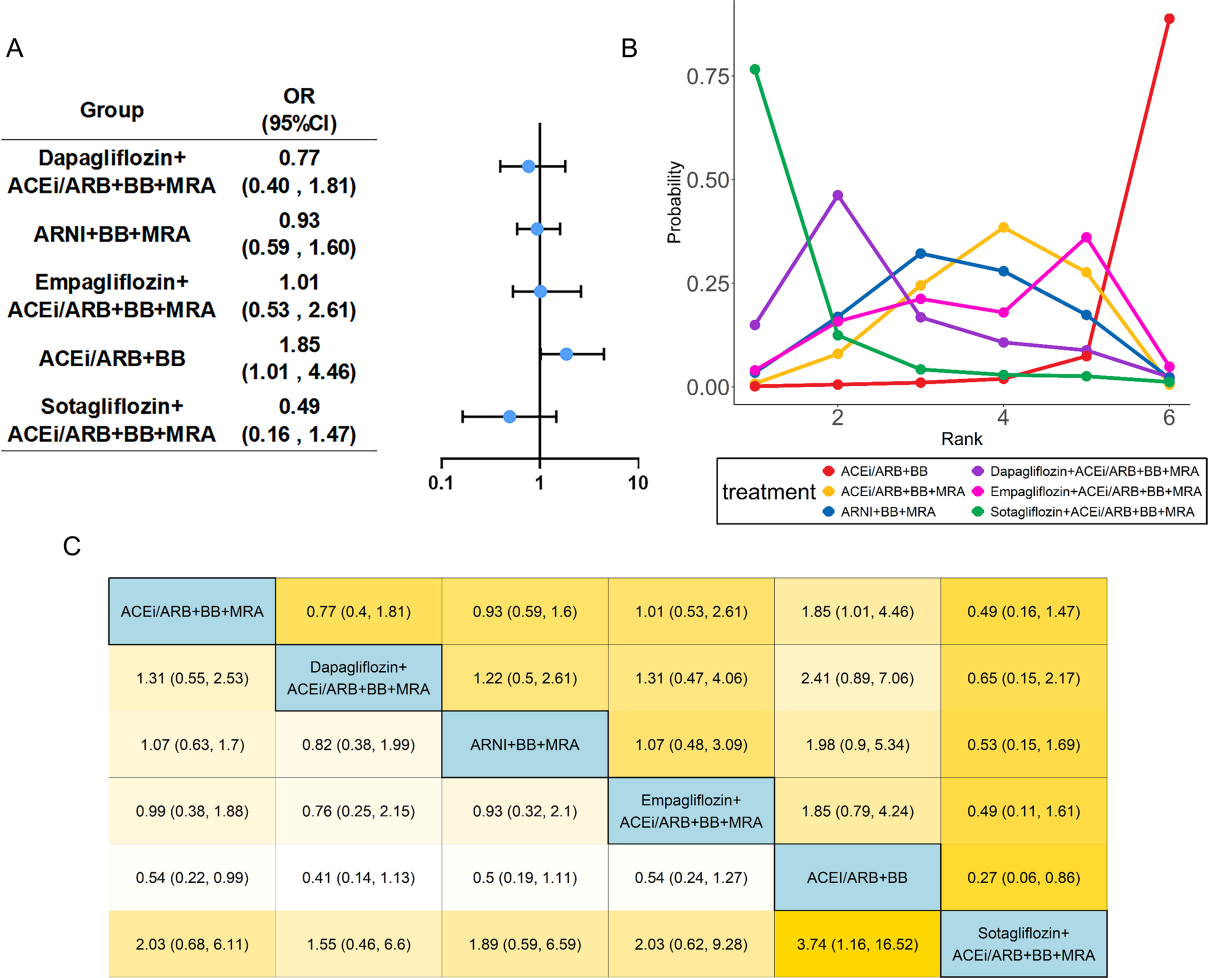
Network meta-analysis of all-cause mortality. The visualization of the results includes **(A)** forest plots, **(B)** rank probability plot, and **(C)** league table. Abbreviations as in **Figure 2**.

Nineteen studies (13–24, 26–28, 30, 31, 33, 34) evaluated all-cause mortality, including 24,030 patients with HFrEF and six treatments. Specific group information of combination medication was shown in a network map (**Supplementary Figure S2B**). Compared with the common control group, Dapagliflozin combined with ACEi/ARB+BB+MRA exhibited an OR of 0.826 (95% CI: 0.694 to 0.984) for reducing the all-cause mortality. Similarly, the regimen of ARNI+BB+MRA showed a protective effect, with an OR of 0.83 (95% CI: 0.75 to 0.92). In contrast, the ACEi/ARB+BB+MRA regimen (OR:1.31, 95%CI:1.07 to 1.61) was associated with an increased risk of the all-cause mortality (**Figure 4**). Based on SUCRA (**Table 1**), the ranking of regimens by efficacy in reducing the all-cause mortality was as follows: Dapagliflozin-based quadruple therapy (76.8%) > ARNI+BB+MRA (76.6%) > Sotagliflozin-based quadruple therapy (67.1%) > Empagliflozin-based quadruple therapy (52.3%) > ACEi/ARB+BB+MRA (26.9%) > ACEi/ARB+BB (0.5%). Heterogeneity testing yielded an *I^2^* of approximately 0, indicating minimal heterogeneity; thus, a fixed effects model was employed for this analysis (**Supplementary Table S2**).

### 6MWD

Eight studies (13, 15, 18–20, 24, 25, 28) evaluated 6MWD, including 1,838 patients with HFrEF and four treatments. Specific group information was shown in a network map with no closed loop (**Supplementary Figure S2C**). For the 6MWD, all active regimens showed point estimates favoring improvement compared to common control. The mean difference (MD) for ARNI+BB+MRA was 37.56 meters (95% CI: -20.32 to 98.53), and for Empagliflozin-based quadruple therapy was 16.45 meters (95% CI: -40.91 to 73.88). Although the confidence intervals are wide and cross the null, the point estimate for ARNI-based therapy (37.56 meters) approaches the range of the minimal clinically important difference (MCID) for 6MWD in HFrEF, which is typically considered to be around 30–40 meters (**Supplementary Figure S3**).

### KCCQ-TSS/KCCQ-CS

Seven studies (13, 17–20, 30, 31) mentioned KCCQ-TSS, encompassing 6,104 patients with HFrEF and five therapeutic regimens. The network plot showed detailed group information with one closed loop (**Supplementary Figure S2D**). Compared with the common control group, Dapagliflozin-based quadruple therapy was associated with the greatest improvement in KCCQ-TSS (MD: 2.97 points, 95% CI: 1.90 to 4.04) (**Supplementary Figure S4**). Although this effect is statistically significant, it is below the commonly cited MCID of 5 points for the KCCQ-TSS, suggesting a modest effect on symptom burden. In contrast, for KCCQ-CS, the changes observed with Empagliflozin (MD: 1.70) and ARNI (MD: 1.68) were also statistically significant but similarly below the MCID threshold for clinical summary scores. According to SUCRA (**Table 1**), Dapagliflozin-based quadruple therapy ranked first (91.3%).

Five studies (13–15, 31, 34) mentioned KCCQ-CS, including 12,770 patients with HFrEF and four treatments. The network plot (**Supplementary Figure S2E**) showed detailed group information with no closed loop. Compared with the common control group, Empagliflozin+ACEi/ARB+BB+MRA (MD: 1.70, 95%CI: 1.67 to 1.73) and ARNI+BB+MRA (MD: 1.68, 95%CI: 0.71 to 2.65) were able to increase KCCQ-CS score with a statistical difference (**Supplementary Figure S5**). According to SUCRA (**Table 1**), Dapagliflozin+ACEi/ARB+BB+MRA had the highest probability of ranking first in increasing KCCQ-CS score.

### NT-proBNP

**Figure 5:**
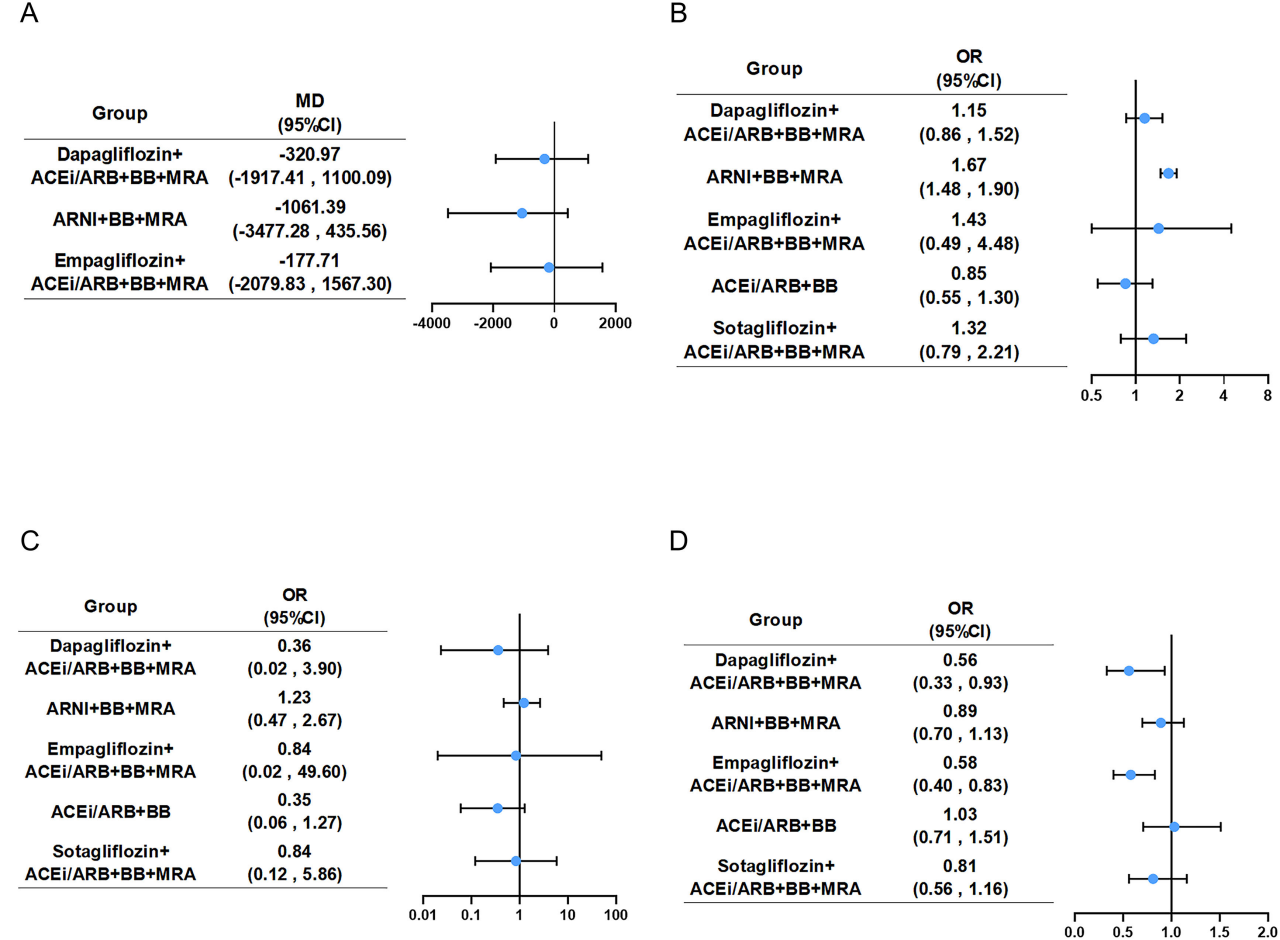
Forest plot for four safety endpoints. Compared with the ACEi/ARB+BB+MRA, five combination groups showed in forest plot of **(A)** NT-proBNP, **(B)** hypotension, **(C)** hyperkalemia and **(D)** renal adverse events. Abbreviations as in **Figure 2**. Graphic summary of NMA on pharmacotherapies for HFrEF, highlighting key findings and clinical implications. ACEi, angiotensin-converting enzyme inhibitor; ARB, angiotensin receptor blocker; ARNI, angiotensin receptor neprilysin inhibitor; BB, beta-blocker; CV, cardiovascular; Dapa, Dapagliflozin; Empa, Empagliflozin; LVEF, left ventricular ejection fraction; MRA, mineral receptor antagonist; NMA, network meta-analysis; Sota, Sotagliflozin; SUCRA, surface under the cumulative ranking curve.

Seven studies (15, 16, 20, 28, 29, 31, 32) evaluated the level of NT-proBNP, including 5,544 patients with HFrEF and four treatments. The network map with no closed loop showed group information of combination medication and there were no significant differences between any of the two comparisons (**Figure 5A**, **Supplementary Figures S6A, S7A**). Based on SUCRA (**Table 1**), the ranking of regimens by efficacy in decreasing the level of NT-proBNP was as follows: ARNI+BB+MRA(87.0%) > Dapagliflozin-based quadruple therapy(51.5%) > Empagliflozin-based quadruple therapy(40.4%) > ACEi/ARB + BB + MRA(21.2%, lowest rank).

### Hypotension/hyperkalemia

Thirteen (13–15, 18, 19, 21, 23, 24, 27, 29, 30, 32, 33) studies mentioned hypotension, encompassing 19,995 patients with HFrEF and six therapeutic regimens. The network plot (**Supplementary Figure S6B**) showed detailed group information with one closed loop. Compared with the common control group, only ARNI+BB+MRA (OR: 1.67, 95%CI: 1.48 to 1.90) demonstrated a clear increase in hypotension likelihood among HFrEF patients (**Figure 5B**, **Supplementary Figure S7B**). According to SUCRA (**Table 1**), ACEi/ARB+BB had the highest probability of ranking first in decreasing the risk of hypotension (87.6%). Heterogeneity assessment yielded a minimal *I^2^* and a fixed effects model was used (**Supplementary Table S2**).

Eleven studies (13, 14, 21–24, 27, 29, 30, 32, 33) mentioned hyperkalemia, encompassing 19,232 patients with HFrEF and six therapeutic regimens. The network plot (**Supplementary Figure S6C**) showed detailed group information with no closed loop. None of the regimens demonstrated a statistically significant difference in hyperkalemia risk versus the common control group (**Figure 5C**, **Supplementary Figures S7C, S8C**). According to SUCRA (**Table 1**), ACEi/ARB+BB had the highest probability of ranking first in decreasing the risk of hyperkalemia (79.8%).

### Renal adverse event

Thirteen (14, 15, 17–19, 21, 23, 24, 27, 29, 30, 33, 34) studies evaluated renal adverse event, including 23,276 patients with HFrEF and six treatments. Group information was shown in a network map (**Supplementary Figure S6D**). Compared with the common control group, Dapagliflozin-based quadruple therapy and Empagliflozin-based quadruple therapy were able to lower the incidence rate of renal adverse event (**Figure 5D**, **Supplementary Figures S7D, S8D**). According to SUCRA (**Table 1**), Empagliflozin-based quadruple therapy ranked first (86.9%), followed by Dapagliflozin-based quadruple therapy(86.2%), Sotagliflozin-based quadruple therapy(51.4%), ARNI+BB+MRA(39.8%), ACEi/ARB+BB+MRA(17.4%), with ACEi/ARB+BB last (18.4%).

### Analysis of inconsistency, publication bias and sensitivity

Inconsistency testing was conducted for CV death and hospitalization, all-cause mortality, KCCQ-TSS, hypotension and renal adverse event (**Supplementary Figure S9**). The results showed that every P value exceeded 0.1, indicating that inconsistency was not significant. Furthermore, a node-splitting analysis was conducted to assess the validity of clustering ACEi and ARB. The results showed no significant inconsistency between direct and indirect evidence for this comparison (P>0.1), supporting the appropriateness of combining them into a single node. For the detection of publication bias in the included literature, the P values of Begg’s Test and Egger’s Test were both greater than 0.05 (**Supplementary Table S2**). No evidence of significant publication bias was found. The results of Sensitivity analysis showed that after excluding any single literature, there was no significant fluctuation in the combined effect size and 95% CI, suggesting that the results were robust (**Supplementary Figures S10, S11**). The varying methodological quality of the included trials, as assessed by the risk of bias tool (**Supplementary Figure S1**), must be considered. However, the low statistical heterogeneity and the stability of results in sensitivity analyses provide reassurance regarding the robustness of the primary conclusions.”

## Discussion

This network meta-analysis represents the most comprehensive comparison to date of six SGLT2 inhibitor-based combination regimens across nine efficacy and safety endpoints in patients with HFrEF. Our findings elucidated that both SGLT2i-based quadruple therapy and ARNI-based triple therapy showed significant superiority over traditional triple therapy(ACEi/ARB+BB+MRA) in reducing the composite cardiovascular endpoint (cardiovascular death and hospitalization) and improving health-related quality of life (HRQoL) measures. However, there were important distinctions existing among them in terms of efficacy, safety, and impact on functional status and biomarkers.

One key finding of this study is the consistent benefit observed across the SGLT2 inhibitor class, supporting the concept of a class effect. While the overall class benefit is clear, our analysis, based on indirect comparisons, suggests potential variations in the point estimates and ranking probabilities (SUCRA values) for different outcomes among the individual agents. These observations are exploratory and may generate hypotheses for tailored therapy, but they should be interpreted with caution in the absence of direct comparative evidence. In our analysis, Dapagliflozin had the highest ranking probability (SUCRA) for improving quality of life scores and reducing all-cause mortality. The point estimates for Empagliflozin were numerically more favorable for renal protection, while the data suggested a potentially higher point estimate for hypotension with this agent, a finding that would require confirmation (35). The hypotensive risk is likely due to insufficient neurohormonal compensation. In contrast to conventional diuretics, empagliflozin fails to robustly activate the RAAS during diuresis, thus blunting the vasoconstrictive compensatory mechanism that normally counteracts volume depletion-induced hypotension (36–38). For renal protection, both Dapagliflozin and Empagliflozin exert potent nephroprotective effects. Current consensus suggests that these SGLT2 inhibitors can activate tubuloglomerular feedback to reduce intraglomerular hypertension, alleviate proximal tubular metabolic stress to restore mitochondrial function, and suppress inflammatory and fibrotic pathways such as NLRP3 inflammasome and TGF-β signaling cascades (39, 40). Lastly, Sotagliflozin’s primary advantage is reducing the composite endpoint of CV death and hospitalization, but it performs poorly in three key safety indicators (hypotension, hyperkalemia and renal adverse event) and has the worst all-cause mortality among the three SGLT2i. Unlike Dapagliflozin and Empagliflozin (selective SGLT2 inhibitors), Sotagliflozin inhibits both SGLT1 and SGLT2: SGLT1 inhibition reduces intestinal glucose absorption, and combined with SGLT2-mediated urinary glucose excretion, this dual action rapidly alleviates heart failure hemodynamic burden—driven mainly by rapid osmotic diuresis and reduced intestinal sodium absorption to lower cardiac preload. Nevertheless, SGLT1 inhibition may exacerbate energy negative balance from reduced intestinal glucose absorption and raise diarrhea risk, indirectly impairing long-term survival.

An exploratory subgroup analysis revealed that diabetes severity exerts a significant impact on the efficacy of SGLT2 inhibitor-based combination regimens in patients with HFrEF, with more severe diabetes correlating with superior therapeutic responses. Heterogeneity assessments demonstrated extremely low within-group heterogeneity yet significant between-group heterogeneity, which validates the reliability of the results. However, It is crucial to emphasize that this analysis was limited by a small number of studies in each subgroup and is likely underpowered. Therefore, this finding must be considered hypothesis-generating and interpreted with extreme caution, as it may be spurious. A plausible underlying mechanism is that greater diabetes severity exacerbates superimposed pathophysiological derangements in HFrEF patients, including hyperglycemia-driven volume overload, and the cardiorenal injury vicious cycle. In contrast, the multi-target actions of SGLT2i combinations, encompassing glycosuria-mediated diuresis, and cardiorenal protection, specifically counteract these abnormalities. Nevertheless, caution is warranted regarding potential effect size bias, which may arise from the higher baseline risk profile of severe diabetes subgroup.

The ARNI+BB+MRA group was significantly superior to other combination regimens in improving 6MWD and reducing NT-proBNP levels (SUCRA >80%). In ARNI, sacubitril inhibits neprilysin to reduce natriuretic peptide degradation and raise their circulating levels. Its valsartan component blocks the angiotensin II type 1 receptor to suppress excessive RAAS activation (41, 42). Their synergy could be the reason for the NT-proBNP reduction. Nevertheless, the synergy of these two components, together with the effects of MRA, contributes to the increased hypotension risk observed in the ARNI + BB + MRA regimen. In addition, the possible mechanism behind the 6MWD improvement could be the regimen enhances cardiac output and optimizes peripheral tissue perfusion, effectively boosting patients’ exercise tolerance.

While SUCRA values provide a useful hierarchy for the relative performance of regimens, the absolute magnitudes of treatment effects are paramount for clinical decision-making. Our findings indicate that several regimens, notably Dapagliflozin-based therapy and ARNI-based therapy, demonstrated not only favorable rankings but also statistically significant and clinically meaningful reductions in hard endpoints like all-cause mortality (ORs: 0.83). An odds ratio of 0.83 translates to a relative risk reduction of approximately 17%, which is substantial in the context of HFrEF where annual mortality rates are high. For patient-reported outcomes such as the KCCQ, the observed improvements, while statistically significant, were generally of a magnitude below established MCID thresholds. This highlights that while these therapies provide measurable benefits, the effect on quality of life in the short to medium term may be modest for the average patient. The interpretation of the composite endpoint of cardiovascular death and hospitalization is nuanced; for instance, the point estimate for Sotagliflozin is promising (OR 0.49) but requires validation in larger studies due to imprecision. Therefore, the choice of therapy should integrate the SUCRA rankings with a careful appraisal of the precision and clinical relevance of the absolute effect sizes for outcomes that matter most to the individual patient.

Furthermore, translating these findings into real-world practice necessitates careful consideration of vulnerable populations, such as those with advanced chronic kidney disease (CKD) or frailty. Our analysis underscores the renal protective profile of Dapagliflozin and Empagliflozin, supporting their use in patients with comorbid CKD in line with current guidelines (43). However, vigilant assessment of baseline renal function and ongoing monitoring remain essential. Conversely, the hypotension risk associated with ARNI-based therapy warrants cautious, slow uptitration in frail, older, or hypotensive patients. While SGLT2 inhibitor-based regimens may offer enhanced benefit in patients with high diabetes burden, applying intensive combination therapies to frail individuals with multimorbidity, poor nutrition, or polypharmacy requires meticulous individualization. This may involve simplifying regimens, slowing titration, and closely monitoring for volume depletion or electrolyte imbalances. Future studies dedicated to these vulnerable subgroups are imperative to solidify evidence-based management strategies.

The clinical implications of our findings are substantial. First, current international guidelines unanimously recommend the foundational use of quadruple therapy but offer limited guidance on the preferred scenarios of SGLT2i/ARNI (1, 4, 44). Our analysis provides much-needed evidence that could inform future updates to refine these recommendations, particularly by confirming the overall class benefit of SGLT2 inhibitors and highlighting the need for direct comparative studies. Second, The data support a shift from a one-size-fits-all quadruple therapy approach towards nuanced regimen selection. For a patient where mortality reduction is the absolute priority, Dapagliflozin-based quadruple therapy appears compelling. For another highly symptomatic patient, Sotagliflozin’s potential advantage in reducing hospitalizations might be favored, provided close monitoring. In patients prone to hypotension, ARNI-based regimens may require more cautious uptitration. Lastly, for clinicians, our SUCRA rankings provide an evidence-based hierarchy for considering which agent to add next to optimize outcomes based on the patient’s most pressing risk.

Several limitations of this study warrant acknowledgment. First, while we applied strict inclusion criteria to support transitivity, residual confounding from unmeasured effect modifiers cannot be entirely excluded, as with all NMAs. Second, subgroup analyses, particularly for diabetes burden, were limited by the small number of studies in each category, making these findings exploratory. Third, clinical heterogeneity, such as variations in follow-up duration and background therapy, existed among trials, though our random-effects model and sensitivity analyses aimed to address this variability. Fourth, certain regimens (e.g., Sotagliflozin-based therapy) included few trials or patients, resulting in wide confidence intervals and less precise estimates. Fifth, subtle differences in endpoint adjudication across studies may introduce methodological heterogeneity, a common challenge in cardiovascular meta-analyses. Finally, our safety analysis focused on clinically significant events (e.g., renal dysfunction, hyperkalemia, hypotension). Other adverse events like volume depletion or genitourinary infections were not quantitatively synthesized due to inconsistent reporting, though clinicians should remain aware of these class-specific risks.

Future research should be prioritized in several key directions to build upon our findings. First, direct head-to-head trials comparing different SGLT2 inhibitors, as well as SGLT2 inhibitors against ARNI, are essential to validate the efficacy hierarchies suggested by this network meta-analysis. Second, studies investigating long-term outcomes beyond hospitalization, such as quality of life, functional decline, and cost-effectiveness, will provide a more comprehensive understanding of treatment value. Third, the optimal sequence of drug initiation and uptitration within quadruple therapy regimens requires clarification through pragmatic clinical trials. Finally, exploring the integration of these pharmacologic strategies with device-based therapies represents a critical frontier in advancing personalized HFrEF management.

## Conclusion

In conclusion, this network meta-analysis suggests that intensifying guideline-directed medical therapy (GDMT) with SGLT2i or ARNI is associated with improved outcomes in patients with HFrEF. Beyond this general finding, our analysis provides evidence that not all quadruple regimens may be equivalent. The relative efficacy and safety profiles identified in this analysis, particularly the SUCR A rankings, could inform the individualization of therapy. When considering which advanced therapy to add, clinicians might consider the patient’s specific efficacy priorities (mortality reduction vs. hospitalization prevention), susceptibility to specific safety risks (hypotension), comorbidities (diabetes, CKD), and patient-reported outcomes. However, these choices should be primarily guided by direct evidence from clinical trials and official clinical guidelines, as our findings are derived from indirect comparisons. Our results provide an evidence basis for this refined, personalized approach to HFrEF management, which aims to maximize therapeutic benefits while minimizing risks for each patient.

## Data Availability

The raw data supporting the conclusions of this article will be made available by the authors, without undue reservation.
