# Supplementary material for: Comparative efficacy and safety of different SGLT2 inhibitor-based combination strategies in HFrEF: a systematic review and network meta-analysis
Source: Front Endocrinol (Lausanne). 2026 Jan 2;16:1742128. doi: 10.3389/fendo.2025.1742128 (PMC12807906; doi:10.3389/fendo.2025.1742128)
Supplement: Supplementary file 1 [file DataSheet1.docx]

**SUPPLEMENTAL MATERIAL**

**Comparative Efficacy and Safety of Different SGLT2 Inhibitor-Based Combination Strategies in HFrEF: A Systematic Review and Network Meta-Analysis**

Neng Jiang^1^; Yuling Zhang^3^, MD, PhD; Yue Tang^2*^, MD, PhD; Hongmei Tan^1*^, MD, PhD

Affiliations: ^1^School of Medicine, Shenzhen Campus of Sun Yat-Sen University, Shenzhen, Guangdong, China; ^2^Department of Cardiovascular Medicine, The Seventh Affiliated Hospital, Sun Yat-sen University, Shenzhen, Guangdong, China; ^3^Department of Cardiovascular Medicine, Sun Yat-sen Memorial Hospital, Sun Yat-sen University, Guangzhou, Guangdong, China. *These authors are joint corresponding authors.

**Supplemental Methods: Search strategy** 2

**Supplemental Tables** 11

**Supplemental Figures** 14

**Supplemental Methods**

**Search strategy**

The following search strategies were executed on the dates specified. The strategy for PubMed is presented in detail, showcasing the combination of MeSH terms and title/abstract keywords. Strategies for other databases were adapted accordingly using their respective controlled vocabularies and field tags.

1. Peer Review: The strategies were peer-reviewed prior to execution.
2. Filters: A study design filter for randomized controlled trials was applied in all databases where feasible.
3. Adaptation Notes:

- For **Web of Science**, the “Topic” (TS) field was used, and the ‘Preprint Citation Index’ was excluded.
- For **EMBASE**, the search fields were ‘ti,ab,kw’ (title, abstract, author keywords).
- For **Cochrane Library**, the search was performed in CENTRAL using the advanced search interface.

**Database: Pubmed**

**Date: July 10^th^, 2025**

#1 "Heart Failure with Reduced Ejection Fraction"[MeSH Terms] OR "left ventricular systolic dysfunction"[Title/Abstract] OR "chronic systolic heart failure"[Title/Abstract] OR "left-sided cardiac failure"[Title/Abstract] OR "heart failure reduced EF"[Title/Abstract] OR "ventricular dysfunction, left"[Title/Abstract] OR "systolic ventricular dysfunction"[Title/Abstract] OR "heart failure, left ventricular"[Title/Abstract] OR "HFrEF"[Title/Abstract] OR "left ventricular failure"[Title/Abstract] OR "congestive systolic heart failure"[Title/Abstract] OR "heart failure, systolic type"[Title/Abstract] OR "reduced ejection fraction heart failure"[Title/Abstract]

#2 "Sodium-Glucose Transporter 2 inhibitors"[MeSH Terms] OR "Sodium-Glucose Transporter 2 inhibitors"[Title/Abstract] OR "gliflozin class"[Title/Abstract] OR "SGLT-2 inhibitor"[Title/Abstract] OR "SGLT2 inhibitor"[Title/Abstract] OR "sodium glucose co-transporter 2 inhibitor"[Title/Abstract] OR "Farxiga"[Title/Abstract] OR "empagliflozin"[Title/Abstract] OR "Canagliflozin"[Title/Abstract] OR "Sotagliflozin" [Title/Abstract] OR "Jardiance"[Title/Abstract] OR "Forxiga"[Title/Abstract] OR "gliflozin medications"[Title/Abstract] OR "dapagliflozin"[Title/Abstract] OR "sglt2i"[Title/Abstract] OR "BMS-512148"[Title/Abstract] OR "bms 512148"[Title/Abstract] OR "sodium-glucose transporter 2 inhibiting agents"[Title/Abstract] OR "BMS512148"[Title/Abstract]

#3 "neprilysin inhibitor"[Title/Abstract] OR "entresto"[Title/Abstract] OR "sacubitril and valsartan"[Title/Abstract] OR "LCZ 696"[Title/Abstract] OR "LCZ696"[Title/Abstract] OR "neutral endopeptidase inhibitor"[Title/Abstract] OR "neprilysin"[Title/Abstract] OR "sacubitril-valsartan combination"[Title/Abstract] OR "LCZ-696"[Title/Abstract] OR "Angiotensin Receptor-Neprilysin Inhibitor"[Title/Abstract] OR "ARNI"[Title/Abstract] OR "endopeptidase inhibitor"[Title/Abstract] OR "sacubitril"[Title/Abstract] OR "angiotensin receptor neprilysin inhibitor"[Title/Abstract]

#4 "Angiotensin-Converting Enzyme Inhibitors"[MeSH Terms] OR "Angiotensin-Converting Enzyme Inhibitors"[Title/Abstract] OR "ACE inhibitor agents"[Title/Abstract] OR "kininase II inhibitor drugs"[Title/Abstract] OR "angiotensin-converting enzyme blocking agents"[Title/Abstract] OR "angiotensin converting enzyme antagonist"[Title/Abstract] OR "ACEIs"[Title/Abstract] OR "inhibitors of angiotensin-converting enzyme"[Title/Abstract] OR "ACEis"[Title/Abstract] OR "kininase II antagonists"[Title/Abstract] OR "enzyme inhibitors, angiotensin converting"[Title/Abstract] OR "ACE inhibiting medications"[Title/Abstract] OR "angiotensin converting enzyme inhibitor class"[Title/Abstract] OR "converting enzyme inhibitors, angiotensin"[Title/Abstract] OR "angiotensin I converting enzyme inhibitors"[Title/Abstract] OR "cilazapril"[Title/Abstract] OR "inhibitor, angiotensin-converting enzyme"[Title/Abstract] OR "kininase 2 inhibitors"[Title/Abstract] OR "ACE inhibitor"[Title/Abstract] OR "angiotensin converting enzyme inhibitors"[Title/Abstract] OR "antagonists of angiotensin converting enzyme"[Title/Abstract] OR "delapril"[Title/Abstract] OR "enalapril"[Title/Abstract] OR "lisinopril"[Title/Abstract] OR "imidapril"[Title/Abstract] OR "indolapril"[Title/Abstract] OR "fosinopril"[Title/Abstract] OR "moexipril"[Title/Abstract]

#5 "Angiotensin Receptor Antagonists"[MeSH Terms] OR "ARBs"[Title/Abstract] OR "Angiotensin Receptor Antagonists" OR "valsartan"[Title/Abstract] OR "Angiotensin II Receptor Blockers"[Title/Abstract] OR "olmesartan"[Title/Abstract] OR "angiotensin receptor antagonist agents"[Title/Abstract] OR "losartan"[Title/Abstract] OR "telmisartan"[Title/Abstract] OR "candesartan"[Title/Abstract] OR "irbesartan"[Title/Abstract] OR "angiotensin receptor blocking drugs"[Title/Abstract] OR "angiotensin II type 1 receptor antagonists"[Title/Abstract] OR "eprosartan"[Title/Abstract] OR "embusartan"[Title/Abstract] OR "receptor antagonists, angiotensin II"[Title/Abstract] OR "AT1 receptor antagonists"[Title/Abstract] OR "angiotensin receptor blocker class"[Title/Abstract] OR "blockers of angiotensin receptors"[Title/Abstract]

#6 "Adrenergic beta-Antagonists"[MeSH Terms] OR "beta blockers"[Title/Abstract] OR "beta-adrenergic receptor antagonists"[Title/Abstract] OR "beta-blocking drugs"[Title/Abstract] OR "adrenergic beta-blocking agents"[Title/Abstract] OR "beta-1 adrenergic blockers"[Title/Abstract] OR "beta antagonist medications"[Title/Abstract] OR "beta adrenergic antagonists"[Title/Abstract] OR "receptor blockers, beta-adrenergic"[Title/Abstract] OR "beta-2 adrenergic antagonists"[Title/Abstract] OR "adrenergic beta receptor blockers"[Title/Abstract] OR "beta blocker class"[Title/Abstract] OR "selective beta blockers"[Title/Abstract] OR "nonselective beta-adrenergic antagonists"[Title/Abstract] OR "beta-adrenergic blocking agents"[Title/Abstract] OR "beta receptor antagonists"[Title/Abstract] OR "bisoprolol"[Title/Abstract] OR "beta-adrenoceptor blocking agents"[Title/Abstract] OR "atenolol"[Title/Abstract] OR "nadolol"[Title/Abstract] OR "metoprolol"[Title/Abstract] OR "antagonists, beta-adrenergic"[Title/Abstract] OR "carvedilol"[Title/Abstract] OR "beta adrenoceptor antagonists"[Title/Abstract] OR "adrenergic beta receptor antagonists"[Title/Abstract] OR "nebivolol"[Title/Abstract] OR "beta-blocking agent"[Title/Abstract] OR "labetalol"[Title/Abstract] OR "beta adrenergic receptor antagonists"[Title/Abstract] OR "acebutolol"[Title/Abstract] OR "receptor antagonists, beta-adrenoceptor"[Title/Abstract] OR "beta antagonist agents"[Title/Abstract] OR "adrenergic beta-blocking agent"[Title/Abstract] OR "beta-receptor antagonists"[Title/Abstract] OR "cardioselective beta blockers"[Title/Abstract] OR "beta adrenergic blocking compounds"[Title/Abstract]

#7 "Mineralocorticoid Receptor Antagonists"[MeSH Terms] OR "spironolactone"[Title/Abstract] OR "aldosterone receptor blockers"[Title/Abstract] OR "eplerenone"[Title/Abstract] OR "mineralocorticoid receptor blocking agents"[Title/Abstract] OR "aldosterone antagonists"[Title/Abstract] OR "receptor antagonists, aldosterone"[Title/Abstract] OR "mineralocorticoid blocking drugs"[Title/Abstract] OR "MR antagonists"[Title/Abstract] OR "aldosterone receptor antagonist agents"[Title/Abstract] OR "mineralocorticoid receptor antagonist class"[Title/Abstract] OR "antagonists of mineralocorticoid receptors"[Title/Abstract] OR "mineralocorticoid antagonist medications"[Title/Abstract] OR "aldosterone receptor blocking agents"[Title/Abstract] OR "canrenone"[Title/Abstract] OR "receptor blockers, mineralocorticoid"[Title/Abstract] OR "MRA"[Title/Abstract]

#8 #2 OR #3 OR #4 OR #5 OR #6 OR #7

#9 #8 AND #1

TOTAL 2963 results

**Database: Web of Science**

**Date: July 9^th^, 2025**

#1 TS=(Heart Failure with Reduced Ejection Fraction) OR TS=(left ventricular systolic dysfunction) OR TS=(chronic systolic heart failure) OR TS=(left-sided cardiac failure) OR TS=(heart failure reduced EF) OR TS=(ventricular dysfunction, left) OR TS=(systolic ventricular dysfunction) OR TS=(heart failure, left ventricular) OR TS=(heart failure, systolic type) OR TS=(HFrEF) OR TS=(left ventricular failure) OR TS=(congestive systolic heart failure) OR TS=(reduced ejection fraction heart failure)

#2 TS=(Sodium-Glucose Transporter 2 inhibitors) OR TS=(Sodium-Glucose Transporter 2 inhibitors) OR TS=(gliflozin class) OR TS=(SGLT-2 inhibitor) OR TS=(SGLT2 inhibitor) OR TS=(sodium glucose co-transporter 2 inhibitor) OR TS=(Farxiga) OR TS=(empagliflozin) OR TS=(Canagliflozin) OR TS=(Sotagliflozin) OR TS=(Jardiance) OR TS=(Forxiga) OR TS=(gliflozin medications) OR TS=(dapagliflozin) OR TS=(sglt2i) OR TS=(BMS-512148) OR TS=(sodium-glucose transporter 2 inhibiting agents) OR TS=(bms 512148) OR TS=(BMS512148)

#3 TS=(neprilysin inhibitor) OR TS=(entresto) OR TS=(sacubitril and valsartan) OR TS=(LCZ 696) OR TS=(LCZ696) OR TS=(neutral endopeptidase inhibitor) OR TS=(neprilysin) OR TS=(sacubitril) OR TS=(sacubitril-valsartan combination) OR TS=(LCZ-696) OR TS=(ARNI) OR TS=(Angiotensin Receptor-Neprilysin Inhibitor) OR TS=(endopeptidase inhibitor) OR TS=(angiotensin receptor neprilysin inhibitor)

#4 TS=(Angiotensin-Converting Enzyme Inhibitors) OR TS=(Angiotensin-Converting Enzyme Inhibitors) OR TS=(ACE inhibitor agents) OR TS=(kininase II inhibitor drugs) OR TS=(ACEIs) OR TS=(angiotensin-converting enzyme blocking agents) OR TS=(angiotensin converting enzyme antagonist) OR TS=(inhibitors of angiotensin-converting enzyme) OR TS=(ACEis) OR TS=(kininase II antagonists) OR TS=(enzyme inhibitors, angiotensin converting) OR TS=(ACE inhibiting medications) OR TS=(angiotensin converting enzyme inhibitor class) OR TS=(converting enzyme inhibitors, angiotensin) OR TS=(angiotensin I converting enzyme inhibitors) OR TS=(cilazapril) OR TS=(inhibitor, angiotensin-converting enzyme) OR TS=(kininase 2 inhibitors) OR TS=(ACE inhibitor) OR TS=(angiotensin converting enzyme inhibitors) OR TS=(antagonists of angiotensin converting enzyme) OR TS=(delapril) OR TS=(enalapril) OR TS=(lisinopril) OR TS=(imidapril) OR TS=(indolapril) OR TS=(fosinopril) OR TS=(moexipril)

#5 TS=(Angiotensin Receptor Antagonists) OR TS=(ARBs) OR TS=(Angiotensin Receptor Antagonists) OR TS=(valsartan) OR TS=(Angiotensin II Receptor Blockers) OR TS=(olmesartan) OR TS=(angiotensin receptor antagonist agents) OR TS=(losartan) OR TS=(telmisartan) OR TS=(candesartan) OR TS=(irbesartan) OR TS=(angiotensin receptor blocking drugs) OR TS=(angiotensin II type 1 receptor antagonists) OR TS=(eprosartan) OR TS=(embusartan) OR TS=(receptor antagonists, angiotensin II) OR TS=(AT1 receptor antagonists) OR TS=(angiotensin receptor blocker class) OR TS=(blockers of angiotensin receptors)

#6 TS=(Adrenergic beta-Antagonists) OR TS=(beta blockers) OR TS=(beta-adrenergic receptor antagonists) OR TS=(beta-blocking drugs) OR TS=(adrenergic beta-blocking agents) OR TS=(beta-1 adrenergic blockers) OR TS=(beta antagonist medications) OR TS=(beta adrenergic antagonists) OR TS=(receptor blockers, beta-adrenergic) OR TS=(beta-2 adrenergic antagonists) OR TS=(adrenergic beta receptor blockers) OR TS=(beta blocker class) OR TS=(selective beta blockers) OR TS=(nonselective beta-adrenergic antagonists) OR TS=(beta-adrenergic blocking agents) OR TS=(beta receptor antagonists) OR TS=(bisoprolol) OR TS=(beta-adrenoceptor blocking agents) OR TS=(atenolol) OR TS=(nadolol) OR TS=(metoprolol) OR TS=(antagonists, beta-adrenergic) OR TS=(carvedilol) OR TS=(beta adrenoceptor antagonists) OR TS=(adrenergic beta receptor antagonists) OR TS=(nebivolol) OR TS=(beta-blocking agent) OR TS=(labetalol) OR TS=(beta adrenergic receptor antagonists) OR TS=(acebutolol) OR TS=(receptor antagonists, beta-adrenoceptor) OR TS=(beta antagonist agents) OR TS=(adrenergic beta-blocking agent) OR TS=(beta-receptor antagonists) OR TS=(cardioselective beta blockers) OR TS=(beta adrenergic blocking compounds)

#7 TS=(Mineralocorticoid Receptor Antagonists) OR TS=(spironolactone) OR TS=(aldosterone receptor blockers) OR TS=(eplerenone) OR TS=(mineralocorticoid receptor blocking agents) OR TS=(aldosterone antagonists) OR TS=(receptor antagonists, aldosterone) OR TS=(mineralocorticoid blocking drugs) OR TS=(MR antagonists) OR TS=(aldosterone receptor antagonist agents) OR TS=(mineralocorticoid receptor antagonist class) OR TS=(antagonists of mineralocorticoid receptors) OR TS=(mineralocorticoid antagonist medications) OR TS=(aldosterone receptor blocking agents) OR TS=(canrenone) OR TS=(receptor blockers, mineralocorticoid) OR TS=(MRA)

#8 #2 OR #3 OR #4 OR #5 OR #6 OR #7 and Preprint Citation Index (Exclude-Database)

#9 TS=(random allocation procedure) OR TS=(placebo-controlled trial) OR TS=(double-blind study) OR TS=(randomized trial) OR TS=(prospective clinical study) OR TS=(randomised controlled trial) OR TS=(single-blind trial) OR TS=(random assignment) OR TS=(randomization method) OR TS=(controlled clinical trial) OR TS=(RCTs) OR TS=(randomly assigned) OR TS=(prospective investigation) OR TS=(blinded trial) OR TS=(placebo comparator)

#10 TS=(drug combination therapy) OR TS=(combination drug therapy) OR TS=(combined drug therapy) OR TS=(polypharmacy) OR TS=(polypharmacy) OR TS=(drug combinations) OR TS=(drug combination) OR TS=(combination of drugs) OR TS=(multiple drug therapy) OR TS=(multidrug therapy) OR TS=(multidrug treatment) OR TS=(concomitant drug use) OR TS=(concomitant medication use) OR TS=(concomitant therapy) OR TS=(co-administration of drugs) OR TS=(drug co-administration) OR TS=(co-therapy) OR TS=(dual drug therapy) OR TS=(triple drug therapy) OR TS=(quadruple drug therapy)

#11 #8 AND #1 AND #9 AND #10 and Preprint Citation Index (Exclude-Database)

TOTAL 2848 results

**Database: Embase**

**Date: July 10^th^, 2025**

#1 (Heart Failure with Reduced Ejection Fraction):ti,ab,kw OR (left ventricular systolic dysfunction):ti,ab,kw OR (chronic systolic heart failure):ti,ab,kw OR (left-sided cardiac failure):ti,ab,kw OR (heart failure reduced EF):ti,ab,kw OR (ventricular dysfunction, left):ti,ab,kw OR (systolic ventricular dysfunction):ti,ab,kw OR (heart failure, left ventricular):ti,ab,kw OR (heart failure, systolic type):ti,ab,kw OR (HFrEF):ti,ab,kw OR (left ventricular failure):ti,ab,kw OR (congestive systolic heart failure):ti,ab,kw OR (reduced ejection fraction heart failure):ti,ab,kw

#2 (Sodium-Glucose Transporter 2 inhibitors):ti,ab,kw OR (Sodium-Glucose Transporter 2 inhibitors):ti,ab,kw OR (gliflozin class):ti,ab,kw OR (SGLT-2 inhibitor):ti,ab,kw OR (SGLT2 inhibitor):ti,ab,kw OR (sodium glucose co-transporter 2 inhibitor):ti,ab,kw OR (Farxiga):ti,ab,kw OR (empagliflozin):ti,ab,kw OR (Canagliflozin):ti,ab,kw OR (Sotagliflozin):ti,ab,kw OR (Jardiance):ti,ab,kw OR (Forxiga):ti,ab,kw OR (gliflozin medications):ti,ab,kw OR (dapagliflozin):ti,ab,kw OR (sglt2i):ti,ab,kw OR (BMS-512148):ti,ab,kw OR (sodium-glucose transporter 2 inhibiting agents):ti,ab,kw OR (bms 512148):ti,ab,kw OR (BMS512148):ti,ab,kw

#3 (neprilysin inhibitor):ti,ab,kw OR (entresto):ti,ab,kw OR (sacubitril and valsartan):ti,ab,kw OR (LCZ 696):ti,ab,kw OR (LCZ696):ti,ab,kw OR (neutral endopeptidase inhibitor):ti,ab,kw OR (neprilysin):ti,ab,kw OR (sacubitril):ti,ab,kw OR (sacubitril-valsartan combination):ti,ab,kw OR (LCZ-696):ti,ab,kw OR (ARNI):ti,ab,kw OR (Angiotensin Receptor-Neprilysin Inhibitor):ti,ab,kw OR (endopeptidase inhibitor):ti,ab,kw OR (angiotensin receptor neprilysin inhibitor):ti,ab,kw

#4 (Angiotensin-Converting Enzyme Inhibitors):ti,ab,kw OR (Angiotensin-Converting Enzyme Inhibitors):ti,ab,kw OR (ACE inhibitor agents):ti,ab,kw OR (kininase II inhibitor drugs):ti,ab,kw OR (ACEIs):ti,ab,kw OR (angiotensin-converting enzyme blocking agents):ti,ab,kw OR (angiotensin converting enzyme antagonist):ti,ab,kw OR (inhibitors of angiotensin-converting enzyme):ti,ab,kw OR (ACEis):ti,ab,kw OR (kininase II antagonists):ti,ab,kw OR (enzyme inhibitors, angiotensin converting):ti,ab,kw OR (ACE inhibiting medications):ti,ab,kw OR (angiotensin converting enzyme inhibitor class):ti,ab,kw OR (converting enzyme inhibitors, angiotensin):ti,ab,kw OR (angiotensin I converting enzyme inhibitors):ti,ab,kw OR (cilazapril):ti,ab,kw OR (inhibitor, angiotensin-converting enzyme):ti,ab,kw OR (kininase 2 inhibitors):ti,ab,kw OR (ACE inhibitor):ti,ab,kw OR (angiotensin converting enzyme inhibitors):ti,ab,kw OR (antagonists of angiotensin converting enzyme):ti,ab,kw OR (delapril):ti,ab,kw OR (enalapril):ti,ab,kw OR (lisinopril):ti,ab,kw OR (imidapril):ti,ab,kw OR (indolapril):ti,ab,kw OR (fosinopril):ti,ab,kw OR (moexipril):ti,ab,kw

#5 (Angiotensin Receptor Antagonists):ti,ab,kw OR (ARBs):ti,ab,kw OR (Angiotensin Receptor Antagonists):ti,ab,kw OR (valsartan):ti,ab,kw OR (Angiotensin II Receptor Blockers):ti,ab,kw OR (olmesartan):ti,ab,kw OR (angiotensin receptor antagonist agents):ti,ab,kw OR (losartan):ti,ab,kw OR (telmisartan):ti,ab,kw OR (candesartan):ti,ab,kw OR (irbesartan):ti,ab,kw OR (angiotensin receptor blocking drugs):ti,ab,kw OR (angiotensin II type 1 receptor antagonists):ti,ab,kw OR (eprosartan):ti,ab,kw OR (embusartan):ti,ab,kw OR (receptor antagonists, angiotensin II):ti,ab,kw OR (AT1 receptor antagonists):ti,ab,kw OR (angiotensin receptor blocker class):ti,ab,kw OR (blockers of angiotensin receptors):ti,ab,kw

#6 (Adrenergic beta-Antagonists):ti,ab,kw OR (beta blockers):ti,ab,kw OR (beta-adrenergic receptor antagonists):ti,ab,kw OR (beta-blocking drugs):ti,ab,kw OR (adrenergic beta-blocking agents):ti,ab,kw OR (beta-1 adrenergic blockers):ti,ab,kw OR (beta antagonist medications):ti,ab,kw OR (beta adrenergic antagonists):ti,ab,kw OR (receptor blockers, beta-adrenergic):ti,ab,kw OR (beta-2 adrenergic antagonists):ti,ab,kw OR (adrenergic beta receptor blockers):ti,ab,kw OR (beta blocker class):ti,ab,kw OR (selective beta blockers):ti,ab,kw OR (nonselective beta-adrenergic antagonists):ti,ab,kw OR (beta-adrenergic blocking agents):ti,ab,kw OR (beta receptor antagonists):ti,ab,kw OR (bisoprolol):ti,ab,kw OR (beta-adrenoceptor blocking agents):ti,ab,kw OR (atenolol):ti,ab,kw OR (nadolol):ti,ab,kw OR (metoprolol):ti,ab,kw OR (antagonists, beta-adrenergic):ti,ab,kw OR (carvedilol):ti,ab,kw OR (beta adrenoceptor antagonists):ti,ab,kw OR (adrenergic beta receptor antagonists):ti,ab,kw OR (nebivolol):ti,ab,kw OR (beta-blocking agent):ti,ab,kw OR (labetalol):ti,ab,kw OR (beta adrenergic receptor antagonists):ti,ab,kw OR (acebutolol):ti,ab,kw OR (receptor antagonists, beta-adrenoceptor):ti,ab,kw OR (beta antagonist agents):ti,ab,kw OR (adrenergic beta-blocking agent):ti,ab,kw OR (beta-receptor antagonists):ti,ab,kw OR (cardioselective beta blockers):ti,ab,kw OR (beta adrenergic blocking compounds):ti,ab,kw

#7 (Mineralocorticoid Receptor Antagonists):ti,ab,kw OR (spironolactone):ti,ab,kw OR (aldosterone receptor blockers):ti,ab,kw OR (eplerenone):ti,ab,kw OR (mineralocorticoid receptor blocking agents):ti,ab,kw OR (aldosterone antagonists):ti,ab,kw OR (receptor antagonists, aldosterone):ti,ab,kw OR (mineralocorticoid blocking drugs):ti,ab,kw OR (MR antagonists):ti,ab,kw OR (aldosterone receptor antagonist agents):ti,ab,kw OR (mineralocorticoid receptor antagonist class):ti,ab,kw OR (antagonists of mineralocorticoid receptors):ti,ab,kw OR (mineralocorticoid antagonist medications):ti,ab,kw OR (aldosterone receptor blocking agents):ti,ab,kw OR (canrenone):ti,ab,kw OR (receptor blockers, mineralocorticoid):ti,ab,kw OR (MRA):ti,ab,kw

#8 #2 OR #3 OR #4 OR #5 OR #6 OR #7

#9 (random allocation procedure):ti,ab,kw OR (placebo-controlled trial):ti,ab,kw OR (double-blind study):ti,ab,kw OR (randomized trial):ti,ab,kw OR (prospective clinical study):ti,ab,kw OR (randomised controlled trial):ti,ab,kw OR (single-blind trial):ti,ab,kw OR (random assignment):ti,ab,kw OR (randomization method):ti,ab,kw OR (controlled clinical trial):ti,ab,kw OR (RCTs):ti,ab,kw OR (randomly assigned):ti,ab,kw OR (prospective investigation):ti,ab,kw OR (blinded trial):ti,ab,kw OR (placebo comparator):ti,ab,kw

#10 #1 AND #8 AND #9

TOTAL 3970 results

**Database: Cochrane library**

**Date: July 10^th^, 2025**

ID Search Hits

#1 MeSH descriptor: [Heart Failure, Systolic] explode all trees 371

#2 (Heart Failure with Reduced Ejection Fraction):ti,ab,kw OR (left ventricular systolic dysfunction):ti,ab,kw OR (chronic systolic heart failure):ti,ab,kw OR (left-sided cardiac failure):ti,ab,kw OR (heart failure reduced EF):ti,ab,kw OR (ventricular dysfunction, left):ti,ab,kw OR (systolic ventricular dysfunction):ti,ab,kw OR (heart failure, left ventricular):ti,ab,kw OR (heart failure, systolic type):ti,ab,kw OR (HFrEF):ti,ab,kw OR (left ventricular failure):ti,ab,kw OR (congestive systolic heart failure):ti,ab,kw OR (reduced ejection fraction heart failure):ti,ab,kw 18889

#3 MeSH descriptor: [Angiotensin-Converting Enzyme Inhibitors] explode all trees 5196

#4 (Angiotensin-Converting Enzyme Inhibitors):ti,ab,kw OR (Angiotensin-Converting Enzyme Inhibitors):ti,ab,kw OR (ACE inhibitor agents):ti,ab,kw OR (kininase II inhibitor drugs):ti,ab,kw OR (ACEIs):ti,ab,kw OR (angiotensin-converting enzyme blocking agents):ti,ab,kw OR (angiotensin converting enzyme antagonist):ti,ab,kw OR (inhibitors of angiotensin-converting enzyme):ti,ab,kw OR (ACEis):ti,ab,kw OR (kininase II antagonists):ti,ab,kw OR (enzyme inhibitors, angiotensin converting):ti,ab,kw OR (ACE inhibiting medications):ti,ab,kw OR (angiotensin converting enzyme inhibitor class):ti,ab,kw OR (converting enzyme inhibitors, angiotensin):ti,ab,kw OR (angiotensin I converting enzyme inhibitors):ti,ab,kw OR (cilazapril):ti,ab,kw OR (inhibitor, angiotensin-converting enzyme):ti,ab,kw OR (kininase 2 inhibitors):ti,ab,kw OR (ACE inhibitor):ti,ab,kw OR (angiotensin converting enzyme inhibitors):ti,ab,kw OR (antagonists of angiotensin converting enzyme):ti,ab,kw OR (delapril):ti,ab,kw OR (enalapril):ti,ab,kw OR (lisinopril):ti,ab,kw OR (imidapril):ti,ab,kw OR (indolapril):ti,ab,kw OR (fosinopril):ti,ab,kw OR (moexipril):ti,ab,kw 12008

#5 MeSH descriptor: [Angiotensin Receptor Antagonists] explode all trees 3001

#6 (Angiotensin Receptor Antagonists):ti,ab,kw OR (ARBs):ti,ab,kw OR (Angiotensin Receptor Antagonists):ti,ab,kw OR (valsartan):ti,ab,kw OR (Angiotensin II Receptor Blockers):ti,ab,kw OR (olmesartan):ti,ab,kw OR (angiotensin receptor antagonist agents):ti,ab,kw OR (losartan):ti,ab,kw OR (telmisartan):ti,ab,kw OR (candesartan):ti,ab,kw OR (irbesartan):ti,ab,kw OR (angiotensin receptor blocking drugs):ti,ab,kw OR (angiotensin II type 1 receptor antagonists):ti,ab,kw OR (eprosartan):ti,ab,kw OR (embusartan):ti,ab,kw OR (receptor antagonists, angiotensin II):ti,ab,kw OR (AT1 receptor antagonists):ti,ab,kw OR (angiotensin receptor blocker class):ti,ab,kw OR (blockers of angiotensin receptors):ti,ab,kw 12072

#7 MeSH descriptor: [Adrenergic beta-Antagonists] explode all trees 5670

#8 (Adrenergic beta-Antagonists):ti,ab,kw OR (beta blockers):ti,ab,kw OR (beta-adrenergic receptor antagonists):ti,ab,kw OR (beta-blocking drugs):ti,ab,kw OR (adrenergic beta-blocking agents):ti,ab,kw OR (beta-1 adrenergic blockers):ti,ab,kw OR (beta antagonist medications):ti,ab,kw OR (beta adrenergic antagonists):ti,ab,kw OR (receptor blockers, beta-adrenergic):ti,ab,kw OR (beta-2 adrenergic antagonists):ti,ab,kw OR (adrenergic beta receptor blockers):ti,ab,kw OR (beta blocker class):ti,ab,kw OR (selective beta blockers):ti,ab,kw OR (nonselective beta-adrenergic antagonists):ti,ab,kw OR (beta-adrenergic blocking agents):ti,ab,kw OR (beta receptor antagonists):ti,ab,kw OR (bisoprolol):ti,ab,kw OR (beta-adrenoceptor blocking agents):ti,ab,kw OR (atenolol):ti,ab,kw OR (nadolol):ti,ab,kw OR (metoprolol):ti,ab,kw OR (antagonists, beta-adrenergic):ti,ab,kw OR (carvedilol):ti,ab,kw OR (beta adrenoceptor antagonists):ti,ab,kw OR (adrenergic beta receptor antagonists):ti,ab,kw OR (nebivolol):ti,ab,kw OR (beta-blocking agent):ti,ab,kw OR (labetalol):ti,ab,kw OR (beta adrenergic receptor antagonists):ti,ab,kw OR (acebutolol):ti,ab,kw OR (receptor antagonists, beta-adrenoceptor):ti,ab,kw OR (beta antagonist agents):ti,ab,kw OR (adrenergic beta-blocking agent):ti,ab,kw OR (beta-receptor antagonists):ti,ab,kw OR (cardioselective beta blockers):ti,ab,kw OR (beta adrenergic blocking compounds):ti,ab,kw 17978

#9 MeSH descriptor: [Mineralocorticoid Receptor Antagonists] explode all trees 968

#10 (Mineralocorticoid Receptor Antagonists):ti,ab,kw OR (spironolactone):ti,ab,kw OR (aldosterone receptor blockers):ti,ab,kw OR (eplerenone):ti,ab,kw OR (mineralocorticoid receptor blocking agents):ti,ab,kw OR (aldosterone antagonists):ti,ab,kw OR (receptor antagonists, aldosterone):ti,ab,kw OR (mineralocorticoid blocking drugs):ti,ab,kw OR (MR antagonists):ti,ab,kw OR (aldosterone receptor antagonist agents):ti,ab,kw OR (mineralocorticoid receptor antagonist class):ti,ab,kw OR (antagonists of mineralocorticoid receptors):ti,ab,kw OR (mineralocorticoid antagonist medications):ti,ab,kw OR (aldosterone receptor blocking agents):ti,ab,kw OR (canrenone):ti,ab,kw OR (receptor blockers, mineralocorticoid):ti,ab,kw OR (MRA):ti,ab,kw 5394

#11 (random allocation procedure):ti,ab,kw OR (placebo-controlled trial):ti,ab,kw OR (double-blind study):ti,ab,kw OR (randomized trial):ti,ab,kw OR (prospective clinical study):ti,ab,kw OR (randomised controlled trial):ti,ab,kw OR (single-blind trial):ti,ab,kw OR (random assignment):ti,ab,kw OR (randomization method):ti,ab,kw OR (controlled clinical trial):ti,ab,kw OR (RCTs):ti,ab,kw OR (randomly assigned):ti,ab,kw OR (prospective investigation):ti,ab,kw OR (blinded trial):ti,ab,kw OR (placebo comparator):ti,ab,kw 1239288

#12 MeSH descriptor: [Sodium-Glucose Transporter 2 Inhibitors] explode all trees 1200

#13 (Sodium-Glucose Transporter 2 inhibitors):ti,ab,kw OR (Sodium-Glucose Transporter 2 inhibitors):ti,ab,kw OR (gliflozin class):ti,ab,kw OR (SGLT-2 inhibitor):ti,ab,kw OR (SGLT2 inhibitor):ti,ab,kw OR (sodium glucose co-transporter 2 inhibitor):ti,ab,kw OR (Farxiga):ti,ab,kw OR (empagliflozin):ti,ab,kw OR (Canagliflozin):ti,ab,kw OR (Sotagliflozin):ti,ab,kw OR (Jardiance):ti,ab,kw OR (Forxiga):ti,ab,kw OR (gliflozin medications):ti,ab,kw OR (dapagliflozin):ti,ab,kw OR (sglt2i):ti,ab,kw OR (BMS-512148):ti,ab,kw OR (sodium-glucose transporter 2 inhibiting agents):ti,ab,kw OR (bms 512148):ti,ab,kw OR (BMS512148):ti,ab,kw 6604

#14 #3 or #4 or #5 or #6 or #7 or #8 or #9 or #10 or #12 or #13 44619

#15 #1 or #2 18973

#16 #15 and #14 and #11 3919

TOTAL 3919 results

**Supplemental Tables**

Table S1: Detailed Characteristics of Included Studies

| Study | Participants(n) | Intervention | Comparator | Follow up duration  (months) | Mean age  (years) | Mean LVEF(%) | Male(%) | combination medications of interests(%) | | |
| --- | --- | --- | --- | --- | --- | --- | --- | --- | --- | --- |
|  |  |  |  |  |  |  |  | ACEi/ARB/ARNi | BB | MRA |
| Patricia Palau 2022 | 90 | Dapagliflozin + ACEi/ARB + BB + MRA | ACEi/ARB + BB + MRA | 3 | 68.6 | 33.8 | 76.7 | 96.7 | 91.1 | 74.4 |
| Martin Halle 2021 | 201 | ARNI + BB + MRA | ACEi/ARB + BB + MRA | 3 | 66.9 | 31.9 | 81.1 | 100 | 94.5 | 77.1 |
| Scott D. Solomon 2020 | 4236 | Dapagliflozin + ACEi/ARB + BB + MRA | ACEi/ARB + BB + MRA | 24 | 66.4 | 31.4 | 76.1 | 93.2 | 96.2 | 71.5 |
| Scott D. Solomon 2020 | 2371 | ARNI + BB + MRA | ACEi/ARB + BB + MRA | 24 | 66 | 31 | 77 | 93.2 | 96 | 71 |
| Michael E. Nassif 2019 | 263 | Dapagliflozin + ACEi/ARB + BB + MRA | ACEi/ARB + BB + MRA | 3 | 61 | 26 | 73 | 91 | 96 | 61 |
| John J.V. McMurray 2024 | 313 | Dapagliflozin + ACEi/ARB + BB + MRA | ACEi/ARB + BB + MRA | 4 | 69 | 29.5 | 74 | 92 | 95 | 58 |
| William T. Abraham 2021 | 312 | Empagliflozin + ACEi/ARB + BB + MRA | ACEi/ARB + BB + MRA | 4 | 69.5 | 30 | 74.4 | 92 | 94.6 | 58.3 |
| M.Packer 2020 | 3730 | Empagliflozin + ACEi/ARB + BB + MRA | ACEi/ARB + BB + MRA | 4 | 66 | 27.5 | 76 | 88.8 | 94.7 | 71 |
| Faiez Zannad 2011 | 2737 | ACEi/ARB + BB | ACEi/ARB + BB + MRA | 21 | 68.6 | 26.1 | 78 | 94 | 86.7 | / |
| Adriaan A. Voors 2022 | 530 | Empagliflozin + ACEi/ARB + BB + MRA | ACEi/ARB + BB | 3 | 70.5 | 31.5 | 66.2 | 70.2 | 79.5 | 53 |
| Enrico Vizzardi 2014 | 130 | ACEi/ARB + BB | ACEi/ARB + BB + MRA | 41 | 62.2 | 36 | / | 99 | 97 | / |
| João Reis 2022 | 40 | Dapagliflozin + ACEi/ARB + BB + MRA | ACEi/ARB + BB + MRA | 6 | 60.9 | 34 | 82.5 | 100 | 97.5 | 90 |
| Jesper Jensen 2020 | 190 | Empagliflozin + ACEi/ARB + BB + MRA | ACEi/ARB + BB + MRA | 3 | 64 | 30 | 83 | 96 | 95 | 66 |
| Douglas L. Mann 2021 | 335 | ARNI + BB + MRA | ACEi/ARB + BB + MRA | 6 | 59 | 20.1 | 73 | 100 | 78 | 58 |
| Marcelo Rodrigues dos Santos 2021 | 52 | ARNI + BB + MRA | ACEi/ARB + BB + MRA | 6 | 58 | 25 | 71 | 100 | 100 | 85 |
| Massimo F. Piepoli 2020 | 621 | ARNI + BB + MRA | ACEi/ARB + BB + MRA | 3 | 67 | ≤40 | 78.5 | 97.5 | 91.6 | 66.7 |
| J.J.V. McMurray 2014 | 8442 | ARNI + BB + MRA | ACEi/ARB + BB + MRA | 27 | 63.8 | 29.5 | 78 | 100 | 93 | 57 |
| Hiroyuki Tsutsui 2021 | 225 | ARNI + BB + MRA | ACEi/ARB + BB + MRA | 33.9 | 68 | 28 | 86 | 100 | 96 | 60 |
| D.L. Bhatt 2020 | 1222 | Sotagliflozin + ACEi/ARB + BB + MRA | ACEi/ARB + BB + MRA | 9 | 70 | 35 | 66 | 91.5 | 92 | 66 |
| Matthew M.Y. Lee 2021 | 105 | Empagliflozin + ACEi/ARB + BB + MRA | ACEi/ARB + BB + MRA | 9 | 68.7 | 32.5 | 77 | 100 | 96 | 63 |
| Yang Gao 2019 | 120 | ARNI + BB + MRA | ACEi/ARB + BB + MRA | 2 | 70.3 | 30.41 | 44 | 68.3 | 92 | 100 |
| J.J.V.McMurray 2019 | 4744 | Dapagliflozin + ACEi/ARB + BB + MRA | ACEi/ARB + BB + MRA | 24 | 66 | 31 | 77 | 93.2 | 96 | 71 |
| Ying Zhao 2022 | 97 | ARNI + BB + MRA | ACEi/ARB + BB + MRA | 6 | 67.7 | 35 | 44.3 | 100 | 88.7 | 94.8 |

Characteristics of included studies on HFrEF drug therapies, including participant numbers, interventions, comparators, follow-up duration, baseline patient characteristics (age, LVEF, sex), and use of combination medications. ACEi: angiotensin-converting enzyme inhibitor; ARB: angiotensin receptor blocker; ARNI: angiotensin receptor neprilysin inhibitor; BB: beta-blocker; MRA: mineral receptor antagonist.

Table S2: DIC, *I^2^* and Publication Bias Test

|  | 6MWD | CV death and hospitalization | all cause mortality | NT-proBNP | KCCQ-TSS | KCCQ-CS | hypotension | hyperkalemia | renal adverse events |
| --- | --- | --- | --- | --- | --- | --- | --- | --- | --- |
| DIC | 32.11 | 62.22 | 57.86 | 30.25 | 28.54 | 17.50 | 47.24 | 45.34 | 55.23 |
| *I*2 | 8% | 16% | 0% | 20% | 9% | 0% | 5% | 16% | 22% |
| Egger's test bias.P value | 0.092 | 0.273 | 0.200 | 0.140 | 0.250 | 0.586 | 0.975 | 0.666 | 0.866 |
| begg's test bias.*P* value | 1.000 | 0.284 | 0.345 | 0.100 | 0.531 | 0.327 | 0.903 | 0.139 | 0.928 |
| node-splitting.*P* value | 0.45 | 0.37 | 0.89 | 0.91 | 0.56 | 0.38 | 0.63 | 0.52 | 0.49 |

Summary table included DIC, *I^2^* for model selection and two publication bias risk test. 6MWD: 6-minute walk distance; CV: cardiovascular; DIC: Deviance information criterion; KCCQ-CS: Kansas City Cardiomyopathy Questionnaire Clinical Summary Score; KCCQ-TSS: Kansas City Cardiomyopathy Questionnaire Total Symptom Score; NT-proBNP: N-terminal pro-B-type natriuretic peptide.

Table S3: Categories, Refined Definitions, and Key Details of Outcome Measures

| Outcome Measure | Category | Refined Definition |
| --- | --- | --- |
| CV death and hospitalization | Primary Outcome | A composite endpoint encompassing study-documented cardiovascular-related death, and hospitalization attributable to confirmed cardiovascular etiologies |
| All-cause mortality | Key Outcome | Death resulting from any etiology, verified by study-reported mortality documentation |
| 6MWD | Functional Outcome | The distance traversed by a subject within 6 minutes, measured via a standardized corridor test |
| KCCQ-TSS | Patient-Reported Outcome | A validated KCCQ-TSS scale (range: 0–100; higher scores indicate superior symptom status) for evaluating total symptom burden in cardiomyopathy patients |
| KCCQ-CSS | Patient-Reported Outcome | The KCCQ-CSS domain (assessing physical/social limitations; range: 0–100) for generating a clinical summary score in cardiomyopathy patients |
| NT-proBNP | Biomarker Outcome | Serum level of N-terminal pro-B-type natriuretic peptide (a biomarker for cardiac function assessment) |
| Hypotension | Safety Outcome | Systolic blood pressure ≤90 mmHg that requires clinical intervention or medication dosage |
| Hyperkalemia | Safety Outcome | Serum potassium concentration ≥5.5 mmol/L, confirmed by repeated laboratory testing |
| Renal adverse events | Safety Outcome | A composite endpoint including: a sustained decline in estimated glomerular filtration rate (eGFR) of ≥40%; end-stage renal disease (defined as sustained [≥28 days] eGFR <15 mL/min/1.73m², sustained dialysis, or renal transplantation); renal death; severe renal insufficiency; acute kidney injury; renal impairment; renal failure; chronic kidney disease |

6MWD: 6-minute walk distance; CV: cardiovascular; DIC: Deviance information criterion; KCCQ-CS: Kansas City Cardiomyopathy Questionnaire Clinical Summary Score; KCCQ-TSS: Kansas City Cardiomyopathy Questionnaire Total Symptom Score; NT-proBNP: N-terminal pro-B-type natriuretic peptide.

**Supplemental Figures**


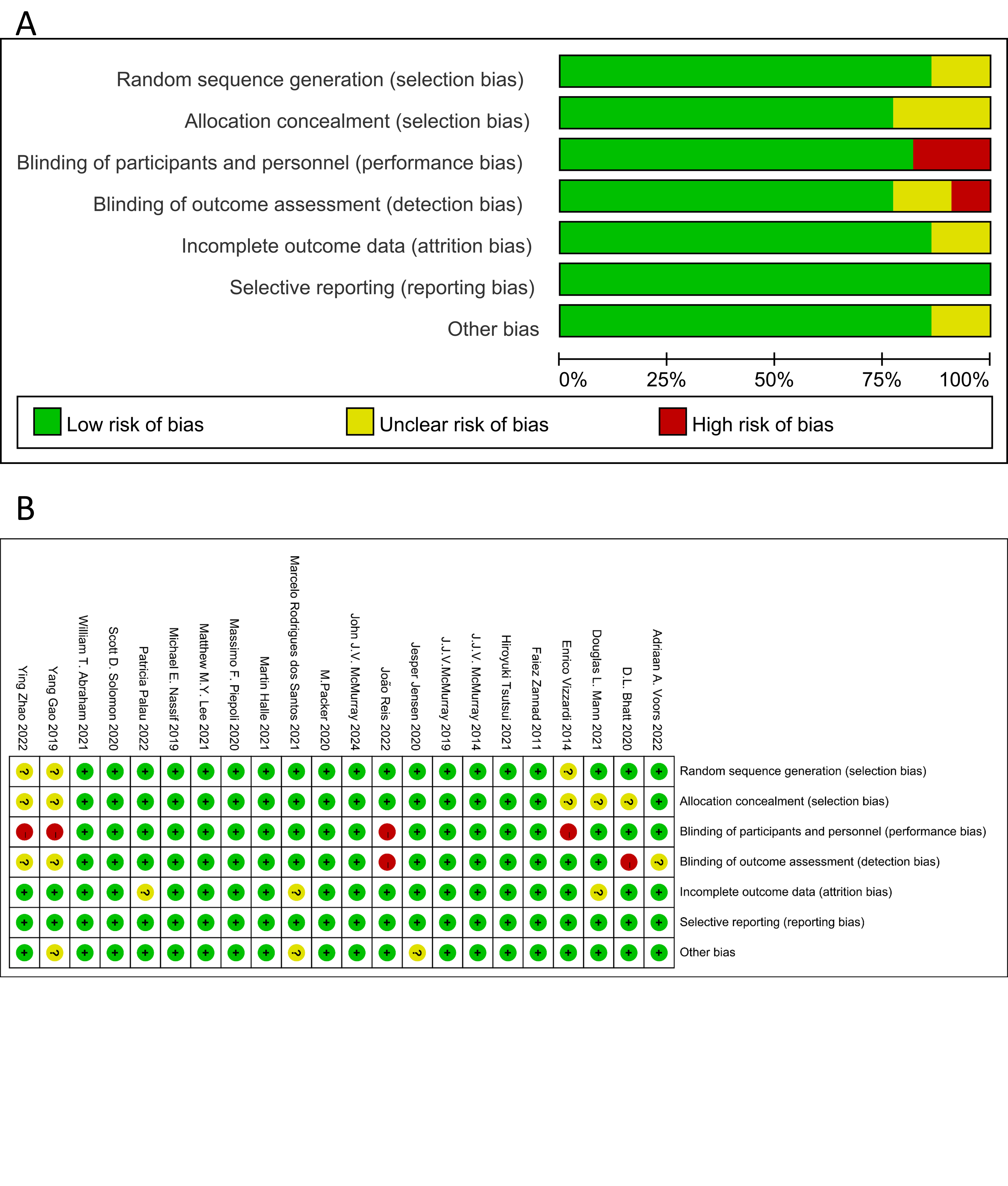
Fig. S1. Risk of Bias Graph and Summary


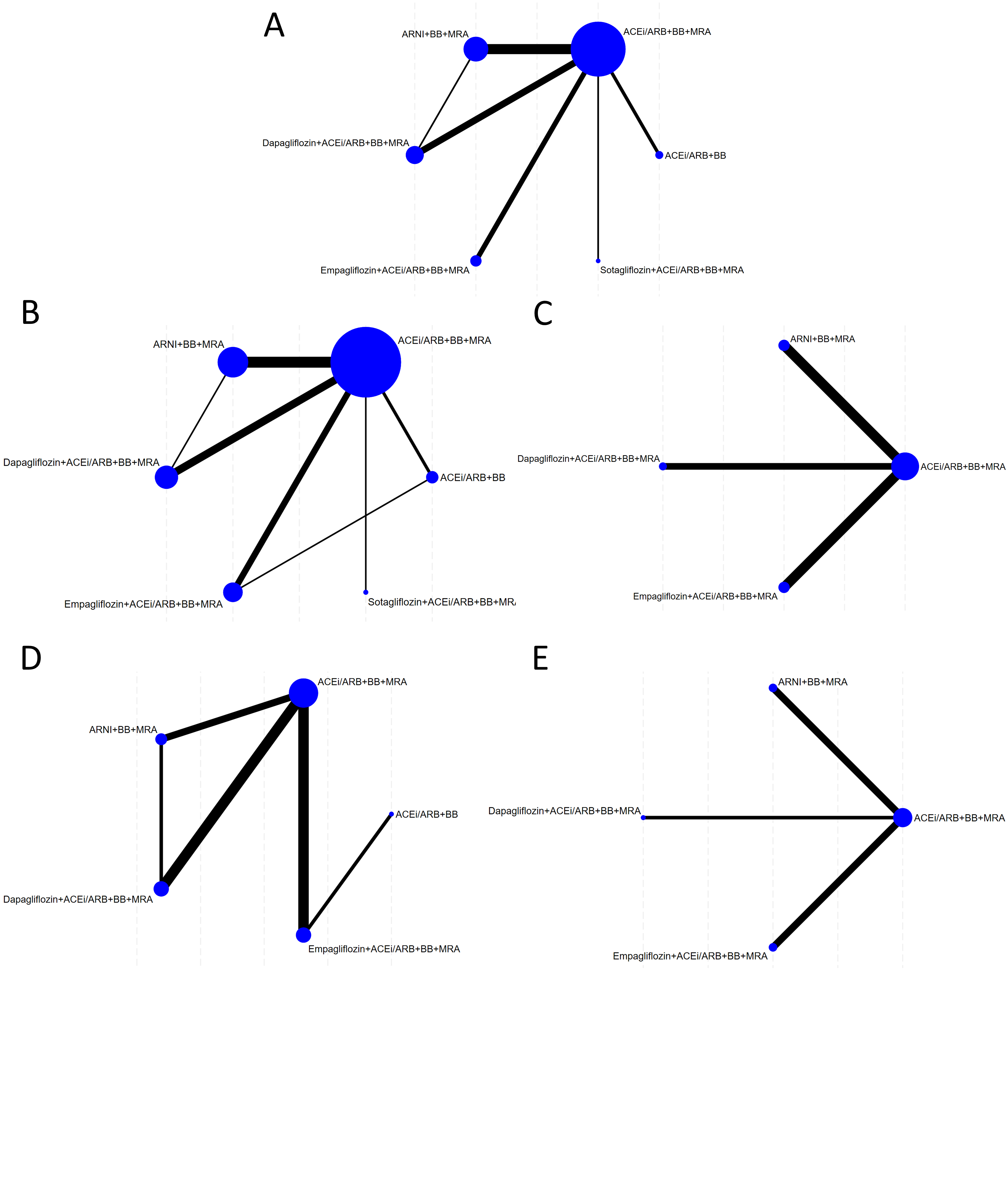


Fig. S2. Network Plots for NMA of Five Outcomes

Network plots for NMA of (A) CV death and hospitalization, (B) all-cause mortality, (C) 6MWD, (D) KCCQ-TSS, and (E) KCCQ-CS. ACEi: angiotensin-converting enzyme inhibitor; ARB: angiotensin receptor blocker; ARNI: angiotensin receptor neprilysin inhibitor; BB: beta-blocker; MRA: mineral receptor antagonist.


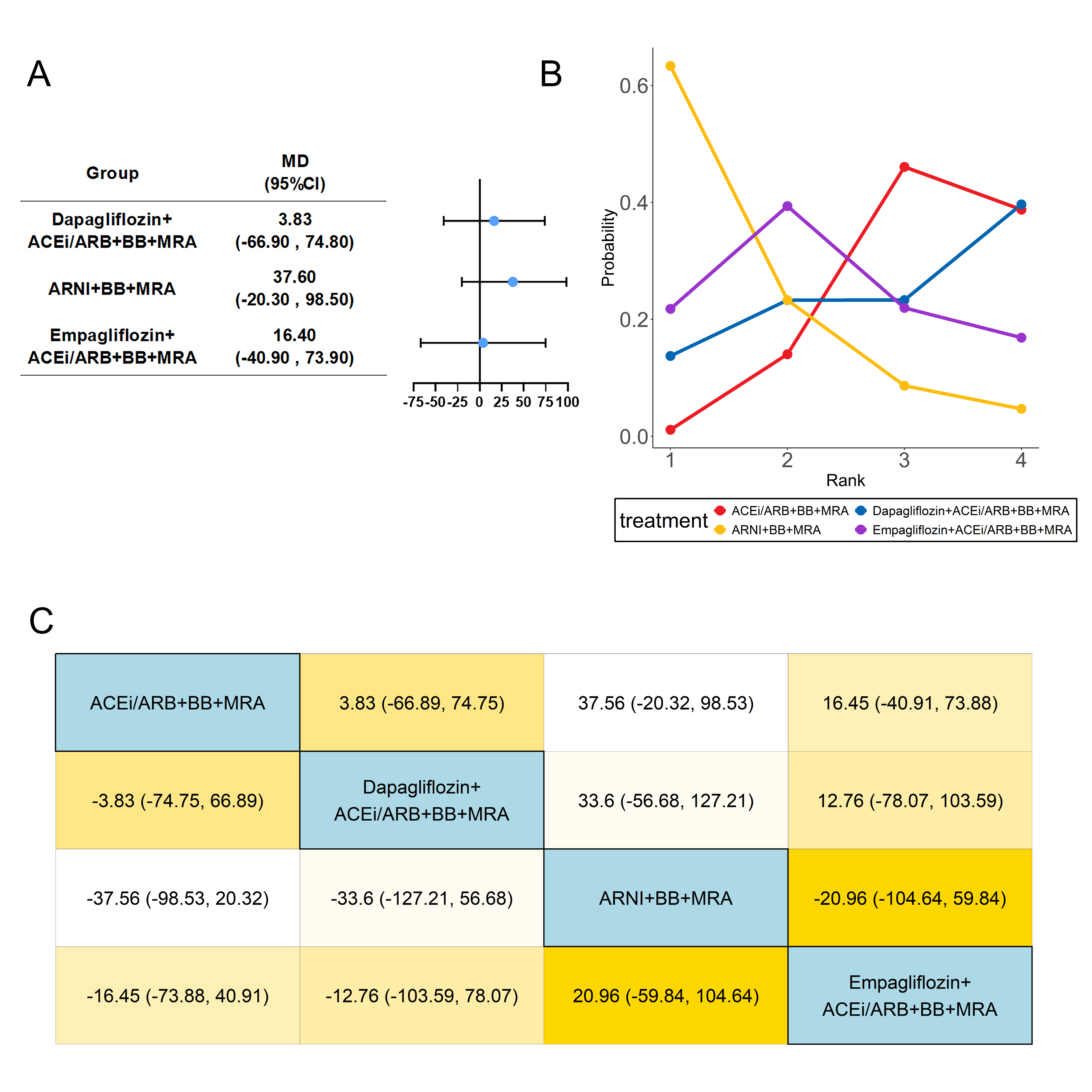


Fig. S3. Network Meta-Analysis of 6MWD

The visualization of the results includes (A) forest plots, (B) rank probability plot, and (C) league table. ACEi: angiotensin-converting enzyme inhibitor; ARB: angiotensin receptor blocker; ARNI: angiotensin receptor neprilysin inhibitor; BB: beta-blocker; MRA: mineral receptor antagonist.


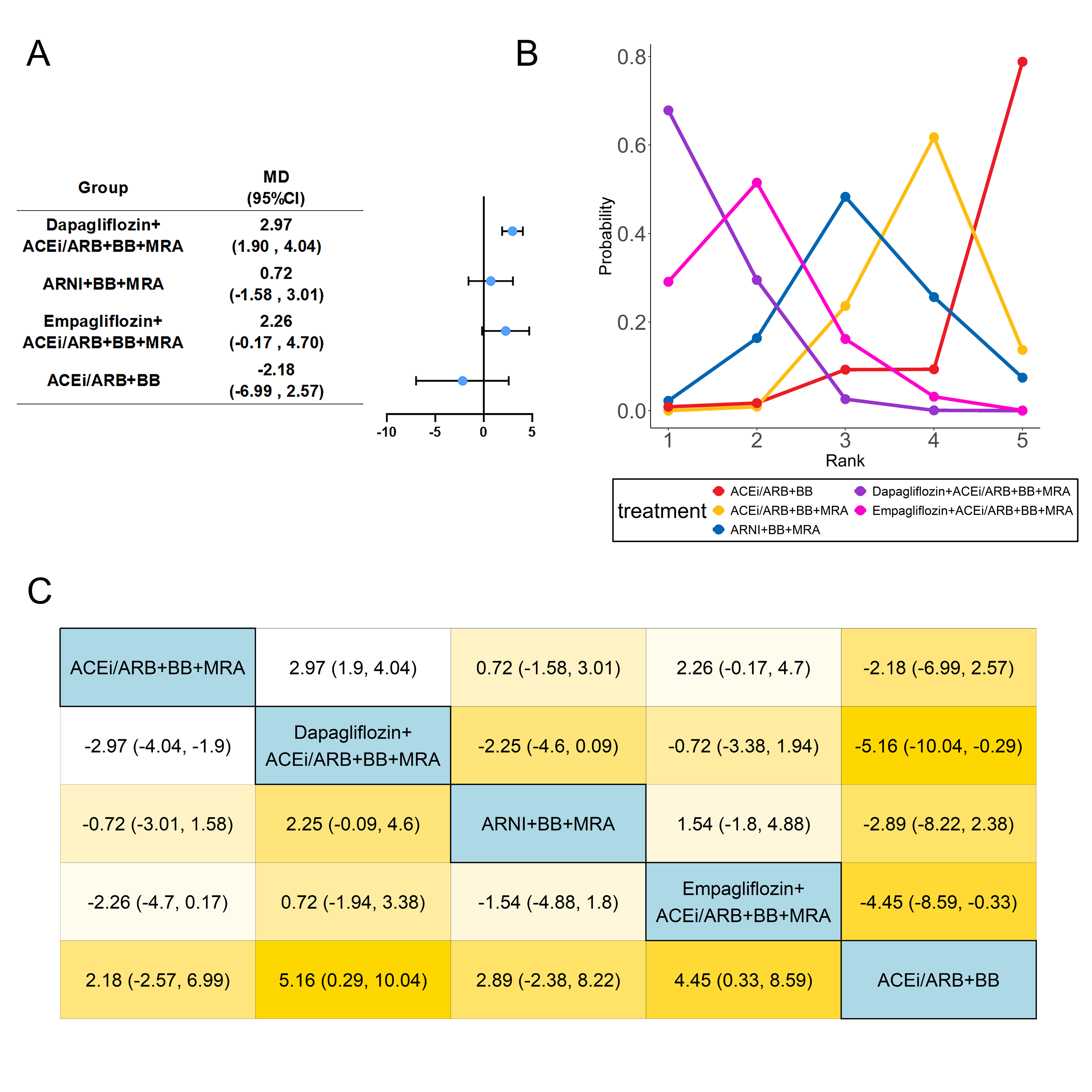


Fig. S4. Network Meta-Analysis of KCCQ-TSS

The visualization of the results includes (A) forest plots, (B) rank probability plot, and (C) league table. ACEi: angiotensin-converting enzyme inhibitor; ARB: angiotensin receptor blocker; ARNI: angiotensin receptor neprilysin inhibitor; BB: beta-blocker; MRA: mineral receptor antagonist.


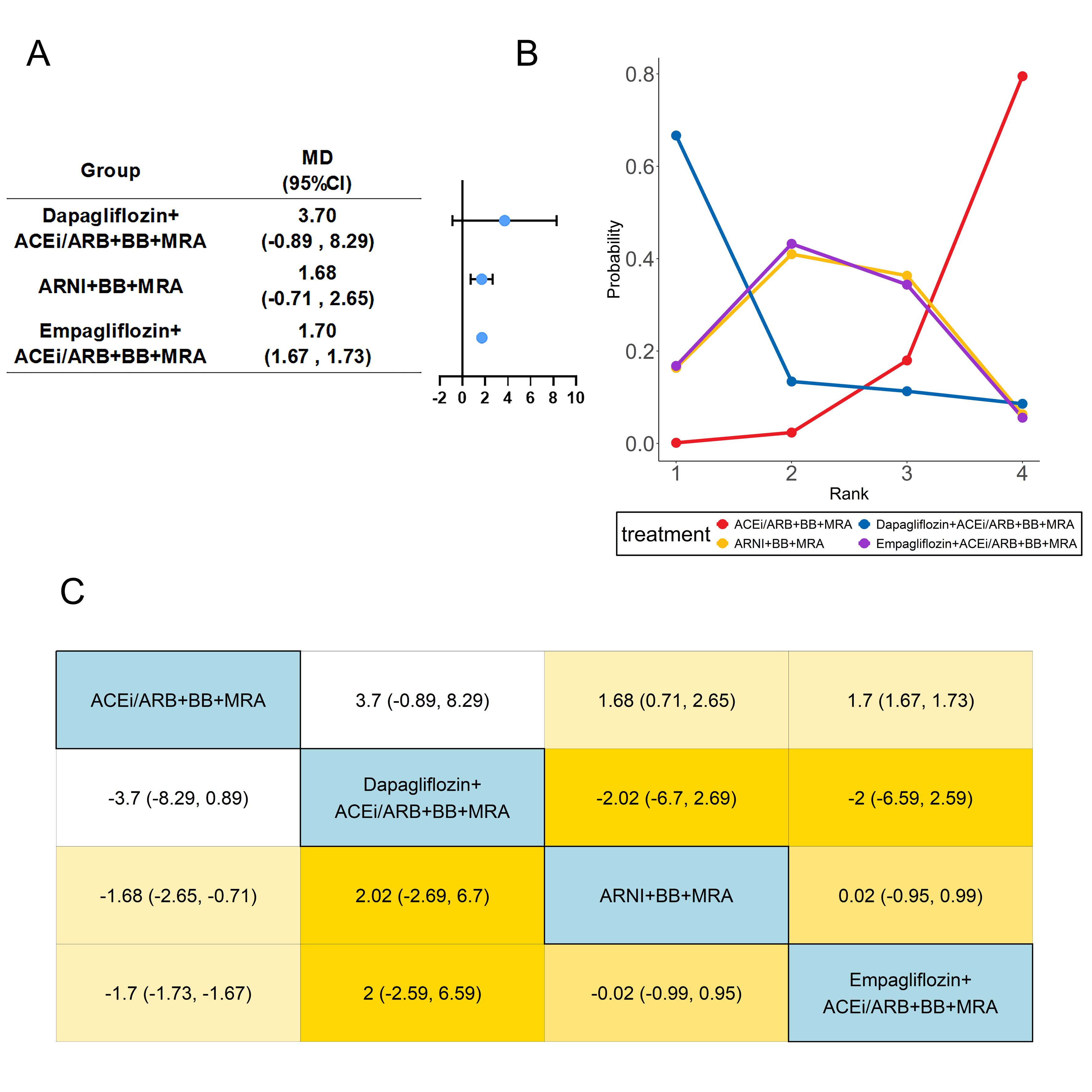


Fig. S5. Network Meta-Analysis of KCCQ-CS

The visualization of the results includes (A) forest plots, (B) rank probability plot, and (C) league table. ACEi: angiotensin-converting enzyme inhibitor; ARB: angiotensin receptor blocker; ARNI: angiotensin receptor neprilysin inhibitor; BB: beta-blocker; MRA: mineral receptor antagonist.


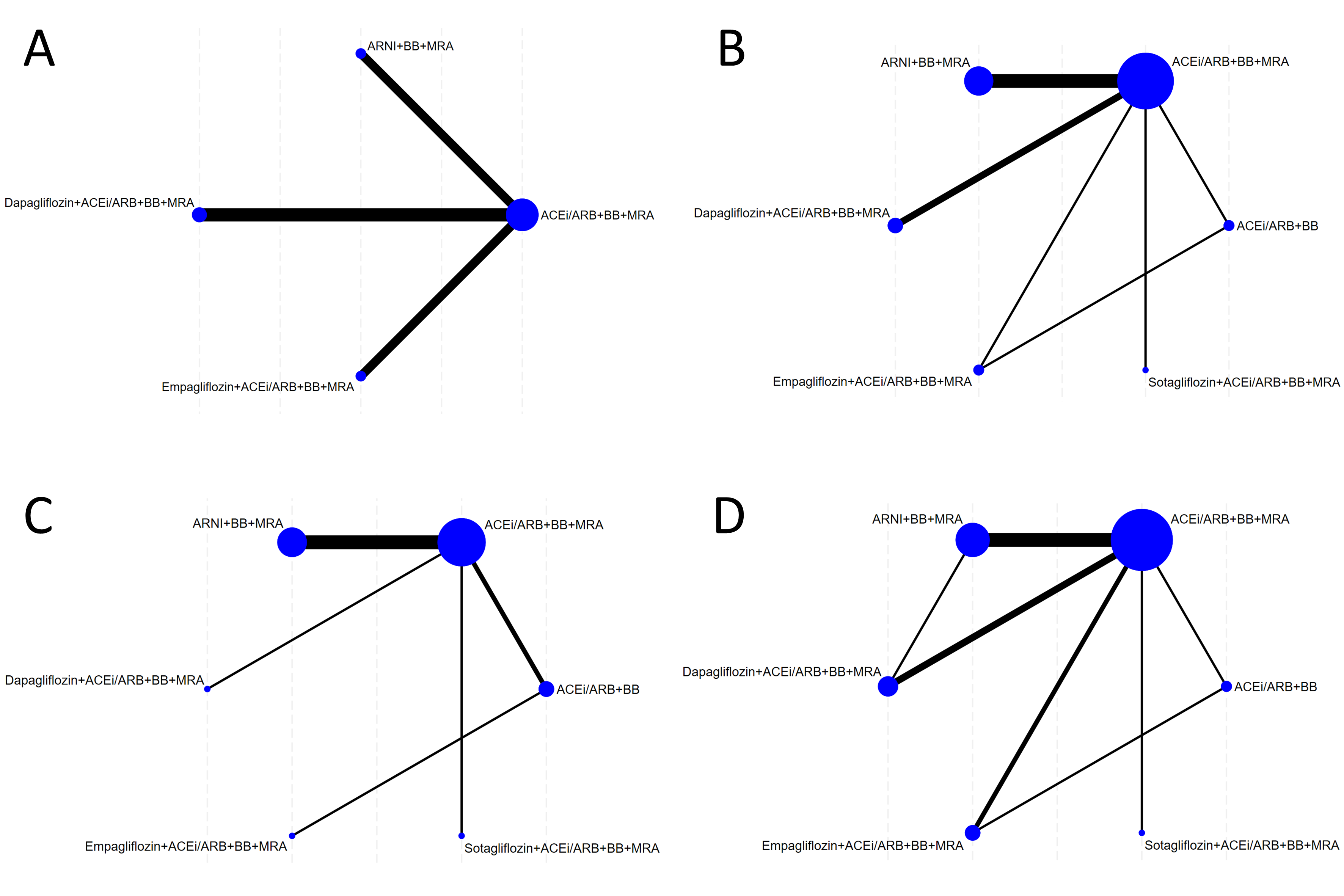


Fig. S6. Network Plots for NMA of Four Outcome

Network plots for NMA of (A) NT-proBNP, (B) hypotension, (C) hyperkalemia, and (D) renal adverse event. ACEi: angiotensin-converting enzyme inhibitor; ARB: angiotensin receptor blocker; ARNI: angiotensin receptor neprilysin inhibitor; BB: beta-blocker; MRA: mineral receptor antagonist.


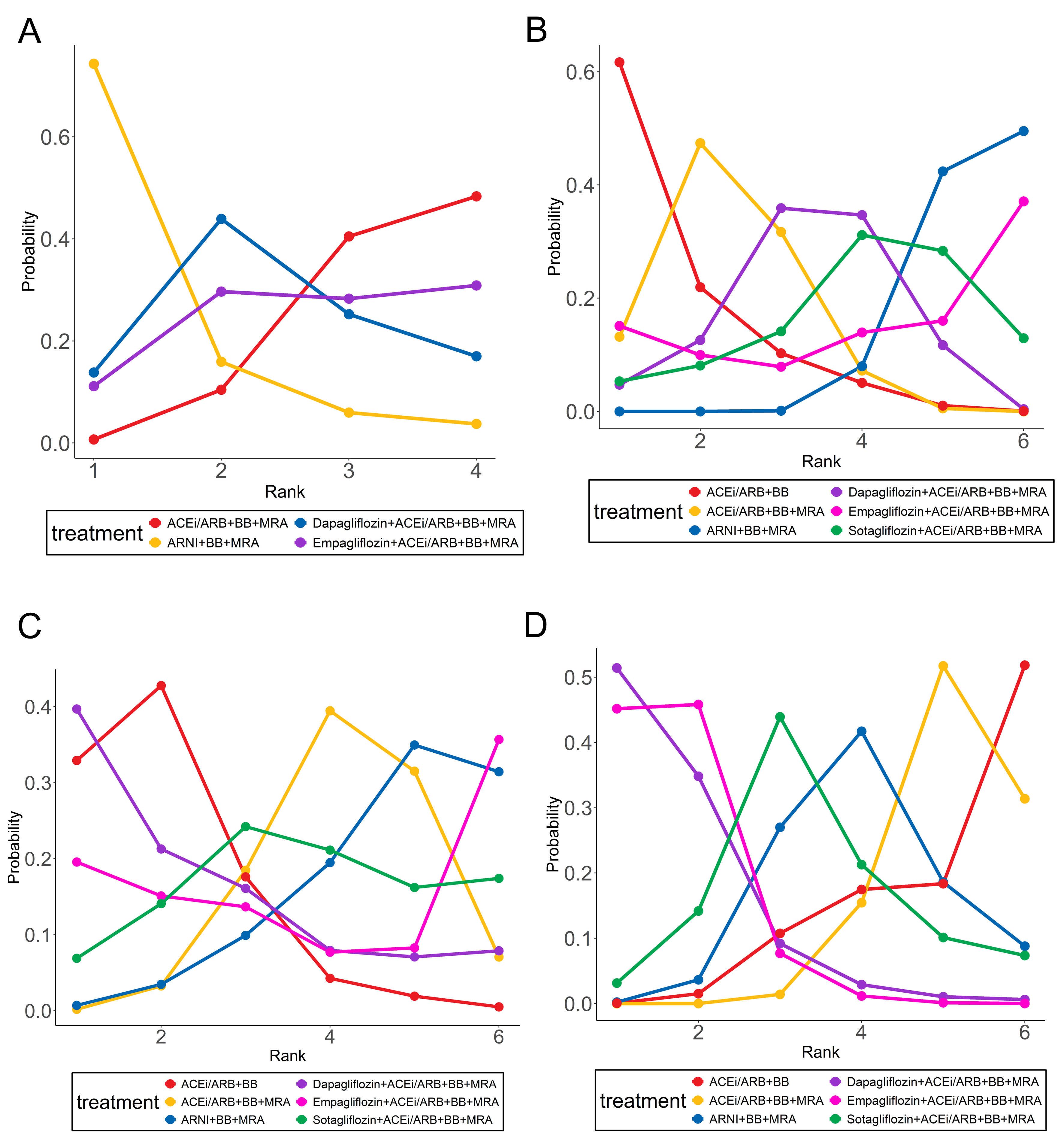


Fig. S7. Rank Probability Plot of Four Safety Endpoints

(A) NT-proBNP, (B) hypotension, (C) hyperkalemia and (D) renal adverse event. ACEi: angiotensin-converting enzyme inhibitor; ARB: angiotensin receptor blocker; ARNI: angiotensin receptor neprilysin inhibitor; BB: beta-blocker; MRA: mineral receptor antagonist.


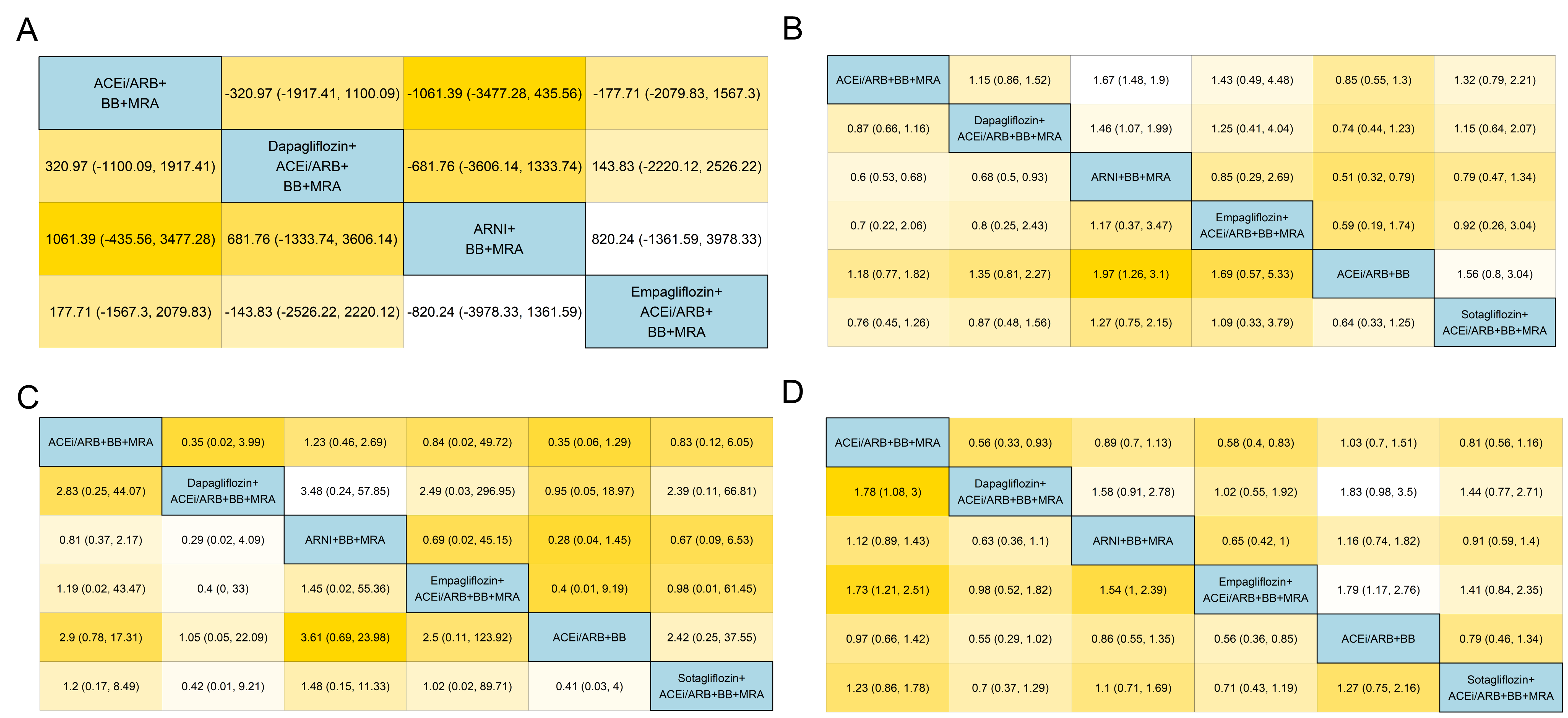


Fig. S8. League Table of Four Safety Endpoints

(A) NT-proBNP, (B) hypotension, (C) hyperkalemia and (D) renal adverse event. ACEi: angiotensin-converting enzyme inhibitor; ARB: angiotensin receptor blocker; ARNI: angiotensin receptor neprilysin inhibitor; BB: beta-blocker; MRA: mineral receptor antagonist.


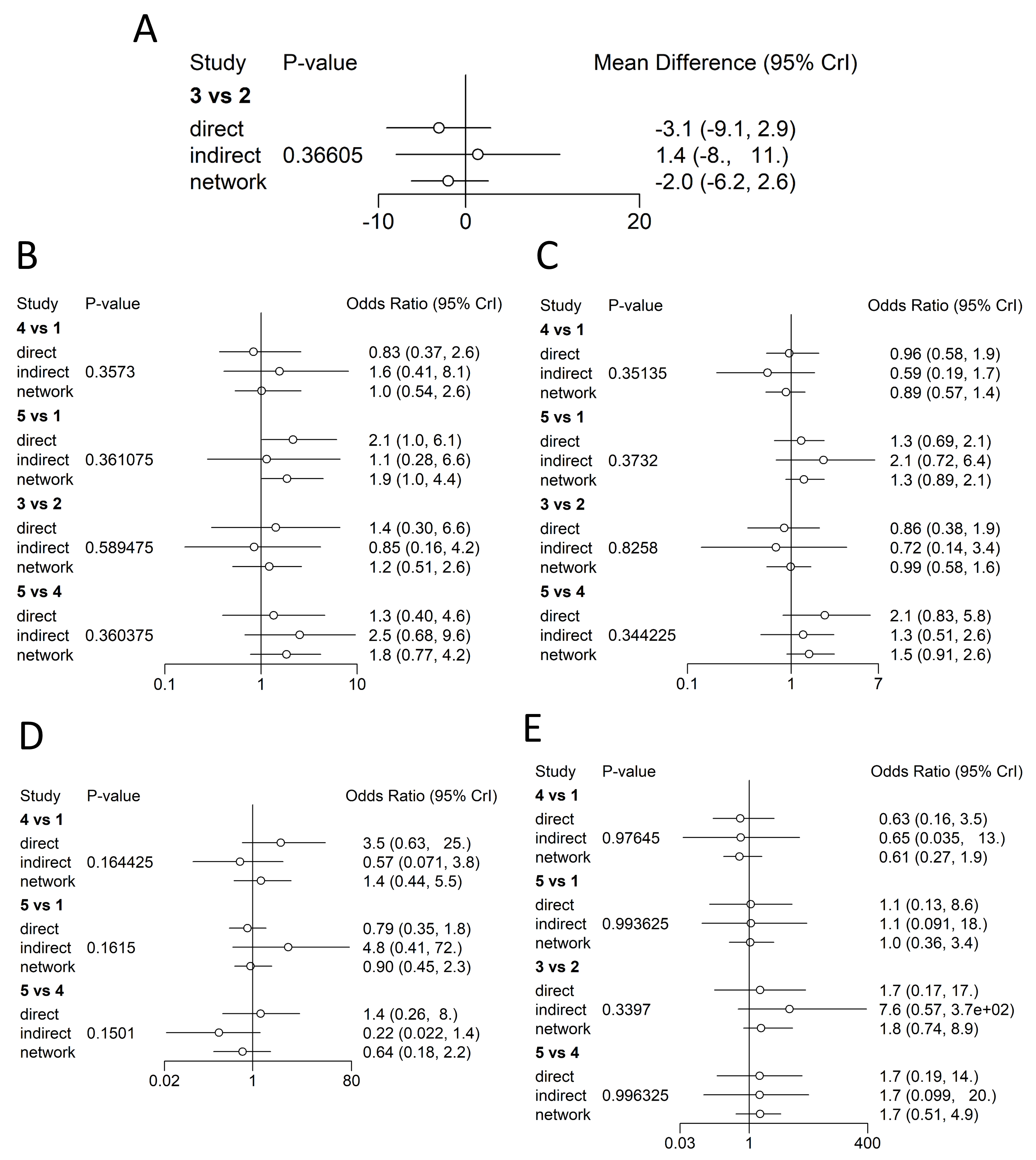


Fig. S9. Forest Plots of Inconsistency Test

Forest plots of inconsistency test for (A) KCCQ-TSS, (B) CV death and hospitalization, (C) all-cause mortality, (D) hypotension, and (E) renal adverse event. 1: ACEi/ARB + BB + MRA; 2: Dapagliflozin + ACEi/ARB + BB + MRA; 3: ARNI + BB + MRA; 4: Empagliflozin + ACEi/ARB + BB + MRA; 5: ACEi/ARB + BB.


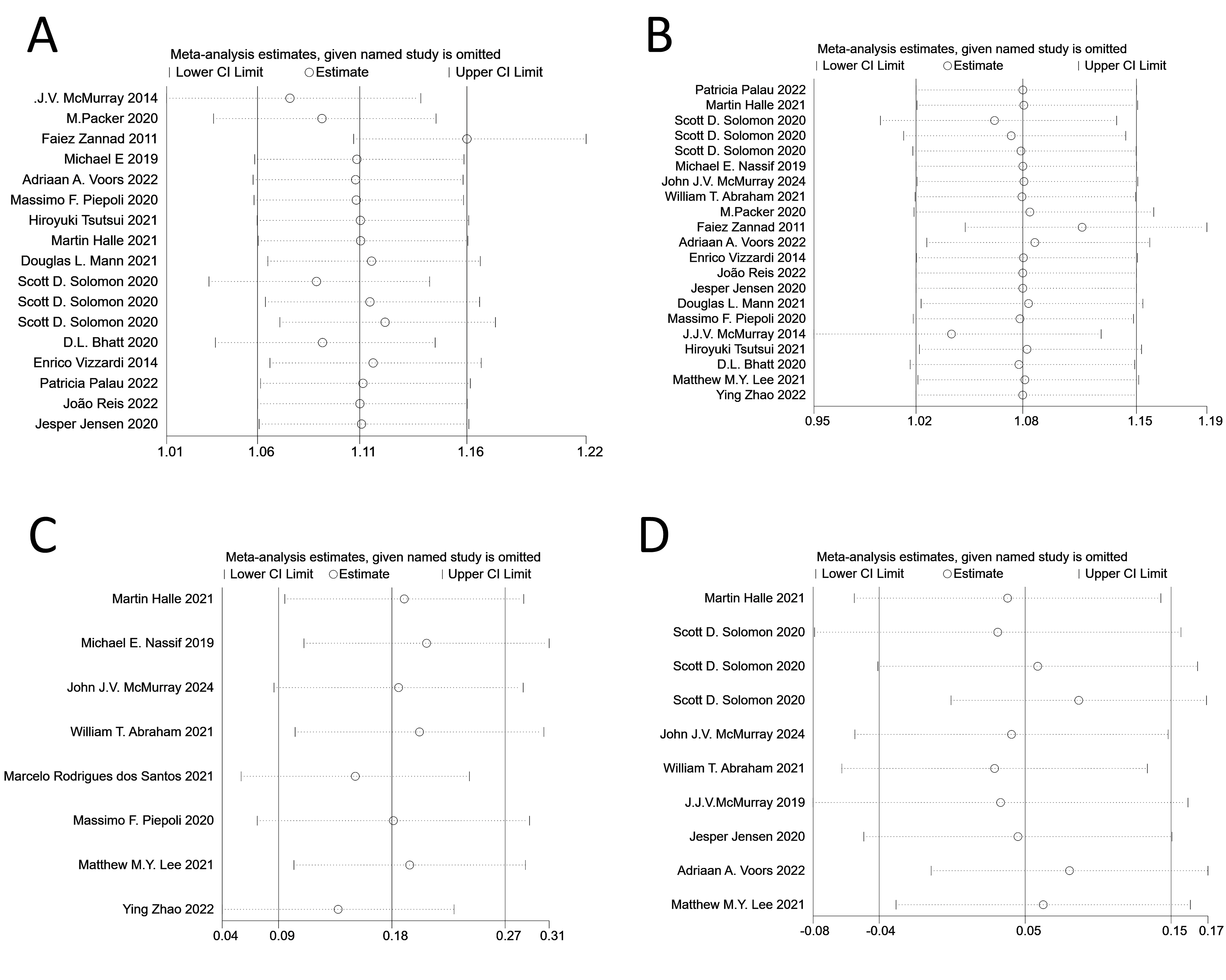
 Fig. S10. Sensitivity Analysis of Four Outcomes

Sensitivity Analysis of (A) CV death and hospitalization, (B) all-cause mortality, (C) 6MWD, and (D) KCCQ-TSS.


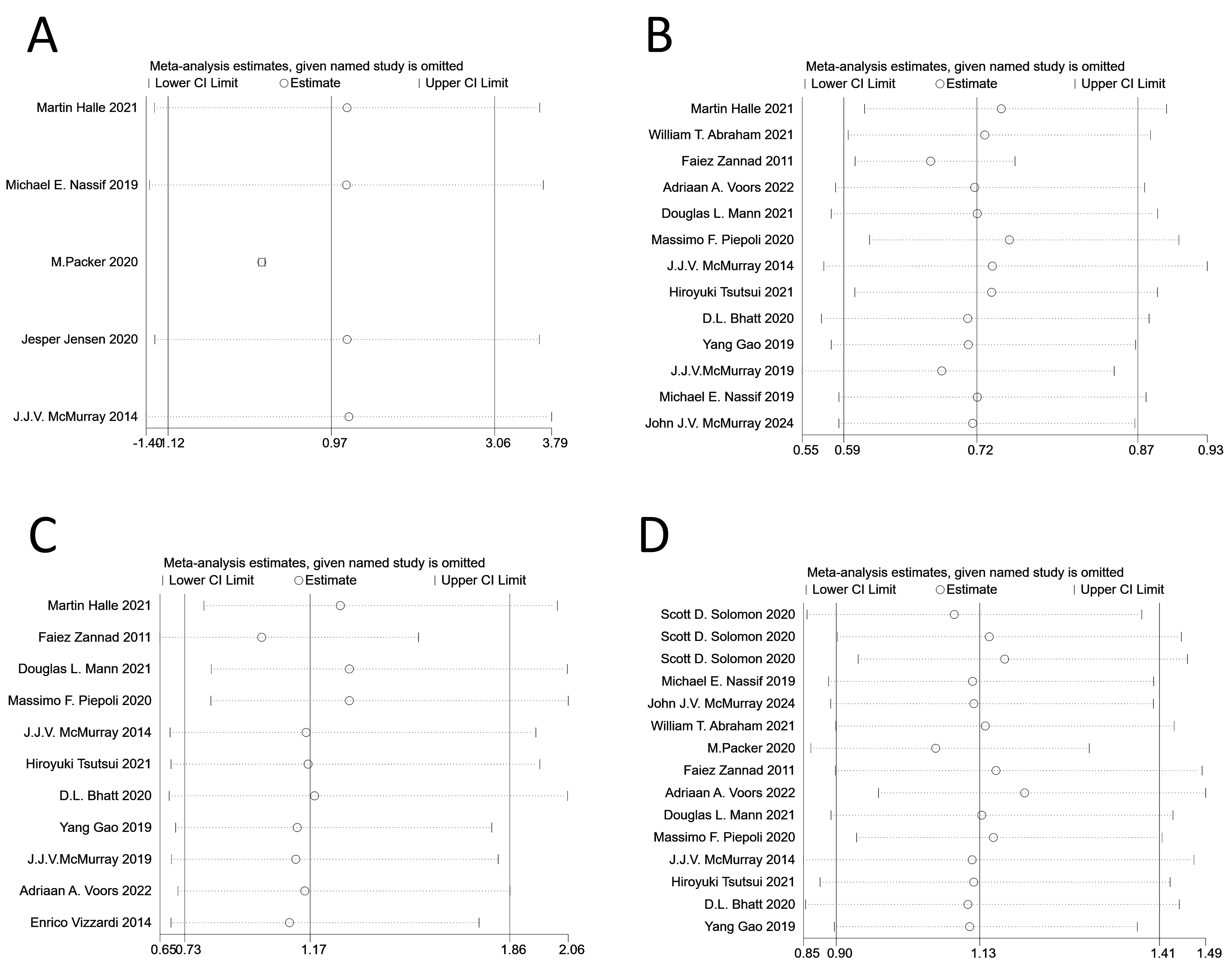


Fig. S11. Sensitivity Analysis of Four Outcomes

Sensitivity Analysis of (A) KCCQ-CS, (B) hypotension, (C) hyperkalemia, and (D) renal adverse event
